# General or Central Obesity and Mortality Among US Hispanic and Latino Adults

**DOI:** 10.1001/jamanetworkopen.2023.51070

**Published:** 2024-01-16

**Authors:** Yanbo Zhang, Guo-Chong Chen, Daniela Sotres-Alvarez, Krista M. Perreira, Martha L. Daviglus, Amber Pirzada, Linda C. Gallo, Maria M. Llabre, Jianwen Cai, Xiaonan Xue, Carmen R. Isasi, Robert Kaplan, Qibin Qi

**Affiliations:** 1 Department of Epidemiology and Population Health, Albert Einstein College of Medicine, Bronx, New York; 2 Department of Nutrition and Food Hygiene, School of Public Health, Medical College of Soochow University, Suzhou, China; 3 Department of Biostatistics, Gillings School of Global Public Health, Chapel Hill, North Carolina; 4 Department of Social Medicine, School of Medicine, University of North Carolina at Chapel Hill, Chapel Hill; 5 Institute for Minority Health Research, University of Illinois, Chicago; 6 Department of Psychology, San Diego State University, San Diego, California; 7 Department of Psychology, University of Miami, Miami, Florida; 8 Division of Public Health Sciences, Fred Hutchinson Cancer Research Center, Seattle, Washington

## Abstract

**Importance:**

The Hispanic and Latino population is the second largest ethnic group in the US, but associations of obesity parameters with mortality in this population remain unclear.

**Objective:**

To investigate the associations of general and central obesity with mortality among US Hispanic and Latino adults.

**Design, Setting, and Participants:**

The Hispanic Community Health Study/Study of Latinos is an ongoing, multicenter, population-based cohort study with a multistage probability sampling method performed in Hispanic and Latino adults aged 18 to 74 years with a baseline between January 1, 2008, and December 31, 2011. Active follow-up for this analyses extended from baseline through February 17, 2022. All analyses accounted for complex survey design (ie, stratification and clustering) and sampling weights to generate estimates representing the noninstitutionalized, 18- to 74-year-old Hispanic or Latino populations from selected communities.

**Exposures:**

Body mass index (BMI; calculated as weight in kilograms divided by height in meters squared), body fat percentage, waist circumference (WC), and waist to hip ratio (WHR).

**Main Outcome and Measure:**

Deaths were ascertained via death certificates, the National Death Index, and active follow-up.

**Results:**

Of 15 773 adults (mean [SE] age, 40.9 [0.3] years; 52.8% female), 686 deaths occurred during a median (IQR) follow-up of 10.0 (9.9-10.2) years. When adjusting for sociodemographic, lifestyle, and family history covariates, hazard ratios (HRs) for mortality were 1.55 (95% CI, 1.08-2.22) for a BMI of 35.0 or greater vs 18.5 to 24.9, 1.22 (95% CI, 0.92-1.64) for the highest vs lowest body fat percentage groups (defined according to sex-, age-, and Hispanic or Latino background–specific BMI distribution), 1.35 (95% CI, 0.98-1.85) for WC greater than 102 cm (men) or 88 cm (women) vs 94 cm (men) or 80 cm (women) or less, and 1.91 (95% CI, 1.28-2.86) for WHR of 0.90 (men) or 0.85 (women) or greater vs less than 0.90 (men) or 0.85 (women). Only WHR was associated with mortality with additional adjustment for major comorbidities (HR, 1.75; 95% CI, 1.17-2.62). The association of WHR with mortality was stronger among women compared with men (*P* = .03 for interaction), and the association between BMI and mortality was stronger among men (*P* = .02 for interaction). The positive association between severe obesity (BMI ≥ 35.0) and mortality was observed only among adults with WHR of 0.90 (men) or 0.85 (women) or greater but not among those with WHR below 0.90 (men) or 0.85 (women) (*P* = .005 for interaction) who had greater hip circumference.

**Conclusions and Relevance:**

In this cohort of US Hispanic and Latino adults, WHR was independently associated with higher all-cause mortality regardless of BMI and prevalent comorbidities. These findings suggest that prioritizing clinical screening and intervention for WHR in this population may be an important public health strategy, with sex-specific strategies potentially being needed.

## Key Points

**Question:**

What are the associations of obesity with mortality among US Hispanic and Latino adults?

**Findings:**

In this population-based cohort study of 15 773 US Hispanic or Latino adults with diverse backgrounds, greater waist to hip ratio was associated with higher mortality regardless of baseline body mass index and comorbidities, whereas severe obesity was only associated with higher mortality among those with unhealthy waist to hip ratio. Sex differences in the associations of body mass index and waist to hip ratio with mortality were observed.

**Meaning:**

These findings suggest that prioritizing clinical screening and intervention of waist to hip ratio may be an important public health strategy among US Hispanic or Latino adults; however, sex-specific strategies might be needed.

## Teaser

This cohort study examines the associations between general and central obesity and mortality among Hispanic or Latino adults in the US.

## Introduction

The Hispanic and Latino population is the second largest and one of the fastest-growing racial or ethnic groups in the US,^1^ with a high obesity prevalence of 45.6% among adults.^2^ Obesity has been linked to increased risks of cardiometabolic diseases, dementia, depression, and cancers,^3^ contributing to more than 5 million deaths and huge disease burdens in 2019 globally.^4^ However, current evidence regarding the association between obesity and mortality is predominantly from White and Asian populations,^5,6,7^ and evidence from Hispanic and Latino individuals, who have different physiologic and sociocultural features,^8,9,10,11^ is sparse and needed desperately.

Of a few publications from Hispanic and Latino populations, most reported lower or similar mortality risks in people with overweight compared with normal weight, and increased mortality risks were observed only in people with severe obesity.^12,13,14,15,16,17,18^ However, no study considered confounding from Hispanic or Latino backgrounds (eg, Cuban and Mexican) and acculturation (ie, the process in which individuals adopt cultural attributes of another culture^19^), which are 2 important determinants of obesity and mortality risks among Hispanic and Latino populations.^11^ Furthermore, few studies controlled for confounding from socioeconomic status, diet, physical activity, and comorbidities. Most evidence considered body mass index (BMI), which measures general obesity (ie, abnormal or excessive fat accumulation),^20^ but BMI cannot distinguish fat from fat-free body mass as body fat percentage can.^7^ Body mass index also cannot evaluate regional body fat distribution, particularly abnormal or excessive abdominal fat accumulation (ie, central obesity),^21^ as waist circumference (WC) and waist to hip ratio (WHR) can. Therefore, we leveraged data from the ongoing population-based Hispanic Community Health Study/Study of Latinos (HCHS/SOL) to investigate the associations of general and central obesity with mortality among US Hispanic and Latino adults.

## Methods

### Study Population

At baseline (January 1, 2008, to December 31, 2011), 16 415 adults aged 18 to 74 years who self-identified as Hispanic or Latino were recruited via a multistage probability sampling method from 4 metropolitan areas in the US: Chicago, Illinois; Miami, Florida; Bronx, New York; and San Diego, California. Participants have diverse backgrounds, including Central and South American, Cuban, Dominican, Mexican, Puerto Rican, and more than 1 ethnicity. Other details are reported in the eMethods in Supplement 1 and previous publications.^22,23,24^ The study was approved by institutional review boards at participating institutions, and all participants provided written informed consent. This study conformed to the Strengthening the Reporting of Observational Studies in Epidemiology (STROBE) reporting guideline.

Among 16 415 participants, we excluded 432 participants with any missing obesity parameter measurement, 125 with a BMI (calculated as weight in kilograms divided by height in meters squared) below 18.5, and 85 who died within the first 2 years of follow-up to reduce the possibility of reverse causation, leaving 15 773 participants in the current analysis (eFigure 1 in Supplement 1).

### Assessment of Obesity Parameters and Covariates

Two general obesity parameters (BMI and body fat percentage) and 2 central obesity parameters (WC and WHR) were measured according to standard procedures at baseline, which are documented in the eMethods in Supplement 1. According to World Health Organization guidelines and previous studies,^20,21,25^ BMI was categorized into 18.5 to 24.9 (normal weight), 25.0 to 29.9 (overweight), 30.0 to 34.9 (moderate obesity), and 35.0 or greater (severe obesity).^20^ Body fat percentage was categorized according to a previous HCHS/SOL study on the basis of sex, age, and Hispanic or Latino background (eMethods in Supplement 1),^25^ with 4 cutoffs corresponding with World Health Organization BMI cutoffs (eTable 1 in Supplement 1): lowest (corresponding to normal weight), second lowest (overweight), second highest (moderate obesity), and highest (severe obesity). Waist circumference (men/women) was categorized as 94/80 cm or less (healthy WC), 95 to 102/81 to 88 cm (intermediate WC), and greater than 102/88 cm (unhealthy WC).^21^ Waist to hip ratio (men/women) was categorized as less than 0.90/0.85 (healthy WHR) and 0.90/0.85 or greater (unhealthy WHR).^21^

Questionnaires were used to collect participants’ demographic (age, sex, Hispanic or Latino background, and marital status), socioeconomic (annual household income, educational level, employment, insurance, length of residence in the US 50 states or Washington, DC, and preferred language), lifestyle (cigarette smoking, alcohol drinking, sleeping time, dietary quality measured by Healthy Eating Index 2010,^26^ and total physical activity levels), and disease (family history of myocardial infarction and diabetes and self-reported prevalent diabetes, cardiovascular disease, cancer [except for nonmelanoma skin cancer], or chronic obstructive pulmonary disease) covariates. Covariate definitions are detailed in the eMethods in Supplement 1.

### Outcome Assessment

All-cause deaths were determined via death certificates (until December 31, 2019), the National Death Index (until December 31, 2019), and proxy reports during annual telephone follow-ups (between December 4, 2017, and February 17, 2022; the median is April 3, 2020). Follow-up time started from baseline until the date of death, loss to follow-up, or last telephone follow-up, whichever occurred first.

### Statistical Analysis

All analyses accounted for complex survey design (ie, stratification and clustering) and sampling weights to generate estimates representing the noninstitutionalized, 18- to 74-year-old Hispanic or Latino populations from selected communities. All analyses were performed using SAS software, version 9.4 (SAS Institute Inc) and SUDAAN, release 11 (Research Triangle Institute) unless otherwise stated. Two-sided *P* < .05 was considered as statistically significant.

Baseline characteristics were described by different WHR and BMI groups. Continuous variables were described as means (SEs), and categorical variables were described as weighted percentages (SEs). Estimates were standardized to the US 2010 Census age distribution. Pearson correlations among obesity parameters were tested.

Age- and sex-standardized mortality rates across obesity groups were estimated. Cox proportional hazards regression models were used to investigate the associations of baseline obesity parameters with mortality. To test the proportional hazards assumption, a product term of follow-up time and each obesity parameter was included, and the assumption was met (*P* > .08). Model 1 adjusted for demographic, socioeconomic, lifestyle, and family history covariates, which were also used in subgroup, sensitivity, and secondary analyses. Model 2 additionally adjusted for prevalent diabetes, cardiovascular disease, cancer, and chronic obstructive pulmonary disease, which were potential mediators between obesity and mortality. Percentages of missing covariate information were less than 5% except for income (6.1%) and sleeping time (5.2%). Missing values for covariates were imputed using the missing indicator approach and sex-specific mean values for categorical and continuous covariates, respectively.^27^ Restricted cubic splines were used to test nonlinear associations, and 5th, 50th, and 95th percentiles were used as knots.^28^

We tested multiplicative and additive interactions between obesity parameters and subgroup covariates—sex, age, Hispanic or Latino background, acculturation (a score considering nativity, years in the US, and language preference),^29^ socioeconomic status, physical activity, and dietary quality (eMethods in Supplement 1)—by introducing a product term of the obesity parameter and covariate into the Cox proportional hazards model and the additive hazards model, respectively. Hazard ratios (HRs) and mortality rate differences (MRDs) comparing different levels of obesity parameters were calculated in different subgroups.^30,31^ Additive hazards models were constructed by the addhazard R package (R Project for Statistical Computing). Several sensitivity analyses were conducted. First, all obesity parameters were categorized into sex-specific quartiles. Second, participants with baseline diabetes, cardiovascular disease, cancer, and chronic obstructive pulmonary disease who could have unintentional weight loss were excluded. Third, participants who died within the first 2 years of follow-up were included. Fourth, we used more stringent criteria to ascertain deaths (eMethods in Supplement 1). Fifth, we included only never-smokers given the associations of smoking with reduced body weight^32^ and multiple disease risks.^4^ Sixth, multiple imputation (5 imputed data sets) was conducted to account for missing covariates.^33^ Seventh, obesity parameters were treated as time-varying variables by including the measurements in 2014 to 2017. Eighth, 1 field center was excluded at a time.

The independent, interaction, and joint associations of general and central obesity with mortality were further examined. Body mass index and WHR were chosen as the indicators for general and central obesity, respectively, given their relatively weaker correlation. First, BMI and WHR were mutually adjusted in the Cox proportional hazards model to test their independent associations. Second, the associations of WHR with mortality by different BMI groups and the associations of BMI with mortality by different WHR groups were examined. To increase statistical power, participants were regrouped into 2 groups according to BMI (<35.0 and ≥35.0). Both multiplicative and additive interactions were tested. Third, the joint association of BMI (<35.0 and ≥35.0) and WHR (<0.90/0.85 and ≥0.90/0.85) with mortality was evaluated.

## Results

### Population Characteristics

Of 15 773 adults (mean [SE] age, 40.9 [0.3] years; 52.8% female and 47.2% male), 25.1% and 21.7% of the population had healthy WHR and normal weight, respectively. The Table and eTable 2 in Supplement 1 show baseline participant characteristics by WHR and BMI groups, respectively. Body fat percentage, BMI, and WC were strongly intercorrelated (*r* range = 0.82-0.91) (eFigure 2 in Supplement 1), whereas WHR was moderately correlated with BMI and body fat percentage (*r* range = 0.33-0.58).

**Table.** Baseline Characteristics of Study Population by Waist to Hip Ratio Groups From the Hispanic Community Health Study/Study of Latinos, 2008-2011^a^

Characteristic	Overall, mean (SE), % (N = 15 773)	Waist to hip ratio	
		<0.90 (men) or <0.85 (women) (n = 3416)	≥0.90 (men) or ≥0.85 (women) (n = 12 357)
Age, mean (SE), y	40.9 (0.3)	34.2 (0.3)	43.4 (0.3)
BMI, mean (SE)	29.5 (0.1)	27.2 (0.2)	30.6 (0.1)
Waist circumference, mean (SE), cm	97.9 (0.2)	87.2 (0.3)	101.8 (0.2)
Waist to hip ratio, mean (SE)	0.9 (0.0)	0.8 (0.0)	1.0 (0.0)
Body fat percentage, mean (SE)	38.9 (0.1)	37.2 (0.2)	40.0 (0.1)
Sex			
Female	52.8 (0.6)	59.7 (1.2)	52.2 (0.7)
Male	47.2 (0.6)	40.3 (1.2)	47.8 (0.7)
Field center			
Bronx, New York	27.6 (1.4)	31.3 (2.1)	26.1 (1.4)
Chicago, Illinois	15.7 (1.0)	10.5 (0.9)	17.2 (1.0)
Miami, Florida	30.7 (2.1)	37.7 (2.6)	28.4 (2.0)
San Diego, California	26.1 (1.7)	20.5 (1.9)	28.3 (1.7)
Hispanic or Latino background			
Central American	7.4 (0.5)	7.3 (0.7)	7.2 (0.5)
Cuban	21.2 (1.7)	25.1 (2.0)	19.7 (1.6)
Dominican	9.6 (0.7)	12.4 (1.0)	8.3 (0.7)
Mexican	37.0 (1.6)	26.2 (1.8)	41.2 (1.6)
Puerto Rican	15.7 (0.8)	18.3 (1.6)	15.0 (0.8)
South American	5.1 (0.3)	6.0 (0.6)	4.7 (0.3)
>1	4.2 (0.3)	4.8 (0.5)	3.9 (0.3)
Marital status			
Married or living with a partner	49.6 (0.8)	45.1 (1.5)	51.5 (0.8)
Separated, divorced, or widow(er)	18.2 (0.5)	20.0 (1.4)	17.8 (0.5)
Single	31.8 (0.6)	34.5 (1.1)	30.4 (0.7)
Yearly household income <$30 000	61.4 (0.9)	59.5 (1.6)	62.2 (1.0)
Educational level			
More than high school or GED	39.1 (0.8)	43.5 (1.5)	38.3 (0.9)
High school graduate or GED	27.1 (0.5)	28.3 (1.1)	26.5 (0.6)
Less than high school or GED	33.5 (0.7)	27.7 (1.4)	34.9 (0.8)
Employment status			
Employed full time	32.5 (0.6)	32.1 (1.1)	32.8 (0.7)
Employed part time	16.3 (0.4)	17.3 (0.9)	15.8 (0.5)
Retired	10.0 (0.3)	8.9 (1.1)	10.1 (0.4)
Unemployed	39.3 (0.7)	40.0 (1.4)	39.4 (0.8)
No current health insurance	47.9 (0.9)	46.6 (1.5)	48.4 (0.9)
Nativity and residence in US			
US born	20.8 (0.7)	19.6 (1.0)	21.4 (0.7)
Non-US born, residence ≥10 y	51.6 (0.7)	48.0 (1.4)	52.9 (0.8)
Non-US born, residence <10 y	27.0 (0.9)	31.6 (1.6)	25.2 (0.9)
Preferred to speak in Spanish	76.8 (0.8)	76.4 (1.6)	76.5 (0.9)
Cigarette smoking			
Never	60.6 (0.6)	66.5 (1.4)	58.8 (0.7)
Former	18.3 (0.5)	14.4 (1.0)	19.3 (0.5)
Current light smoker	10.1 (0.4)	8.7 (0.6)	10.7 (0.5)
Current heavy smoker	10.5 (0.4)	10.1 (0.9)	10.7 (0.5)
Alcohol drinking			
Never	19.3 (0.7)	22.2 (1.4)	18.3 (0.7)
Former	30.1 (0.7)	28.9 (1.5)	30.6 (0.7)
Current moderate drinker	44.5 (0.7)	44.2 (1.4)	44.3 (0.8)
Current heavy drinker	5.9 (0.3)	4.4 (0.5)	6.6 (0.4)
Sleep <6 or >9 h/d	24.1 (0.5)	24.4 (1.4)	24.2 (0.6)
Family history of myocardial infarction	30.0 (0.6)	31.7 (1.3)	29.8 (0.6)
Family history of diabetes	41.0 (0.7)	37.8 (1.5)	42.5 (0.8)
Physical activity			
High	54.8 (0.6)	57.0 (1.4)	54.3 (0.6)
Medium	10.6 (0.3)	10.8 (0.8)	10.4 (0.4)
Low	12.6 (0.4)	11.5 (0.9)	12.7 (0.5)
None	21.4 (0.5)	19.8 (1.2)	21.9 (0.6)
Healthy Eating Index-2010			
Quintile 5	20.8 (0.7)	21.0 (1.5)	20.9 (0.7)
Quintile 4	20.2 (0.5)	20.0 (1.2)	20.1 (0.6)
Quintile 3	21.2 (0.5)	20.2 (1.1)	21.8 (0.6)
Quintile 2	19.3 (0.5)	20.7 (1.1)	18.7 (0.6)
Quintile 1	18.6 (0.6)	18.1 (0.9)	18.5 (0.6)
Prevalent diabetes	17.9 (0.5)	9.1 (0.9)	19.8 (0.5)
Prevalent cardiovascular disease	8.1 (0.3)	5.9 (0.8)	8.6 (0.4)
Prevalent cancer	3.6 (0.2)	3.3 (0.6)	3.8 (0.3)
Prevalent chronic obstructive pulmonary diseases	6.3 (0.3)	7.2 (1.3)	6.2 (0.3)

Abbreviation: GED, General Equivalency Diploma.

^a^ The analysis considered complex and multistage probability sampling (ie, sampling weight, stratification, and clustering), and all estimates (except age) were standardized to the US 2010 Decennial Census age distribution. Data are presented as percentage (SE) unless otherwise indicated.

### Associations of Obesity Parameters With Mortality

**Figure 1.**
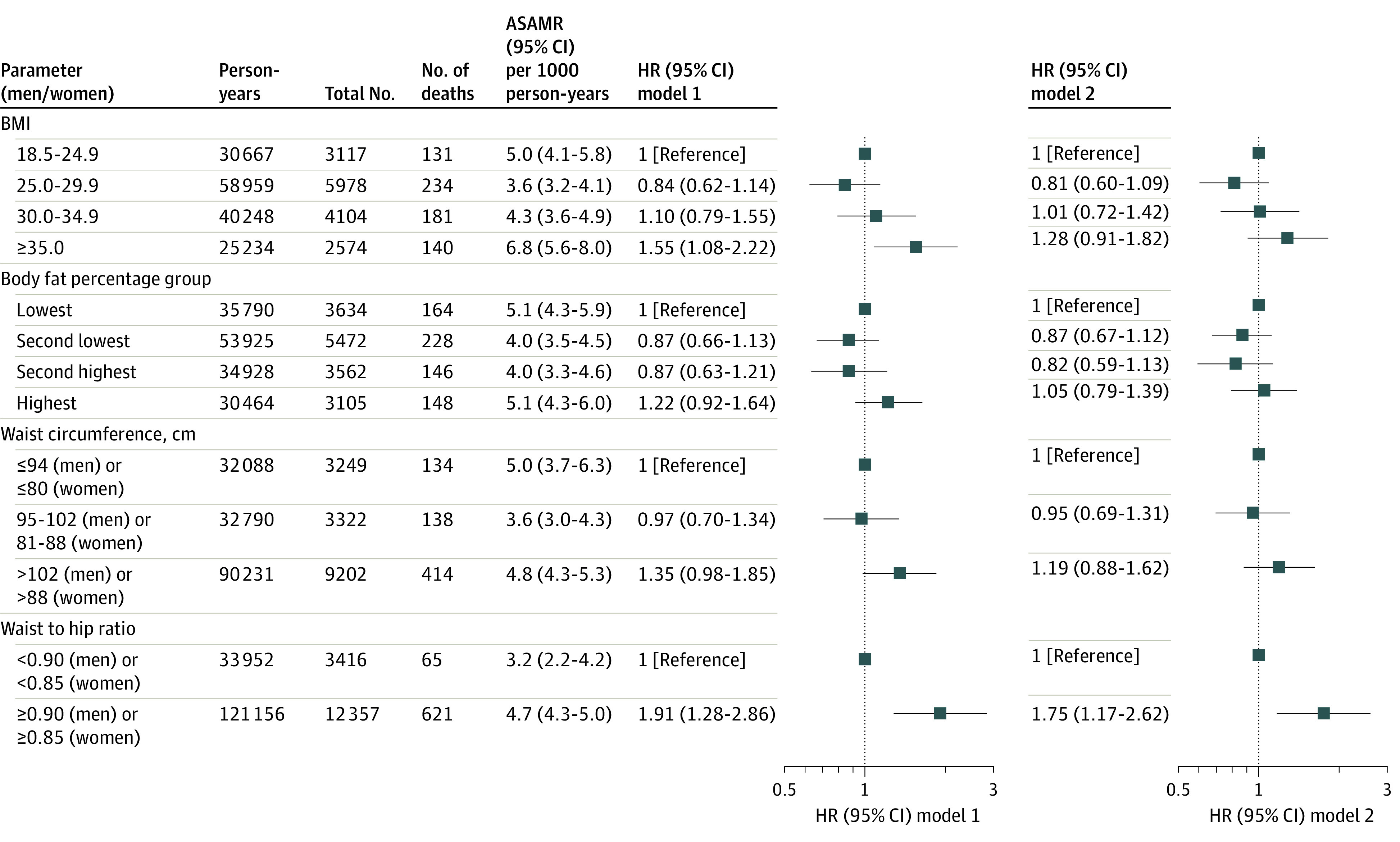
Associations Between Different Obesity Parameters and Mortality, the Hispanic Community Health Study/Study of Latinos (2008-2011) Model 1 was adjusted for age, sex, field center, Hispanic or Latino background, marital status, income, educational level, employment status, insurance, length of stay in the US, preferred language, family history of myocardial infarction and diabetes, cigarette smoking, alcohol drinking, sleeping time, diet, and physical activity. Model 2 was additionally adjusted for prevalent diabetes, cardiovascular disease, cancer, and chronic obstructive pulmonary disease. Body mass index (BMI; calculated as weight in kilograms divided by height in meters squared) was categorized as 18.5 to 24.9 (normal weight), 25.0 to 29.9 (overweight), 30.0 to 34.9 (moderate obesity), and 35.0 or greater (severe obesity). Groups of body fat percentage are defined in eTable 1 in Supplement 1. Waist circumference was categorized as 94 cm (men) or 80 cm (women) or less (healthy waist circumference), 95 to 102 cm (men) or 81 to 88 cm (women) (intermediate waist circumference), and greater than 102 cm (men) or 88 cm (women) (unhealthy waist circumference). Waist to hip ratio was categorized as less than 0.90 (men) or less than 0.85 (women) (healthy waist to hip ratio) and 0.90 (men) or 0.85 (women) or greater (unhealthy waist to hip ratio). ASAMR indicates age- and sex-adjusted mortality rate; HR, hazard ratio.

During a total of 155 108 person-years of follow-up (median [IQR], 10.0 [9.9-10.2] years), 686 deaths were recorded (Figure 1). Age- and sex-adjusted mortality rates per 1000 person-years were 5.0 (95% CI, 4.1-5.8) and 6.8 (95% CI, 5.6-8.0) among normal weight and severe obesity groups, 5.1 (95% CI, 4.3-5.9) and 5.1 (95% CI, 4.3-6.0) among the lowest and highest body fat percentage groups, 5.0 (95% CI, 3.7-6.3) and 4.8 (95% CI, 4.3-5.3) among healthy and unhealthy WC groups, and 3.2 (95% CI, 2.2-4.2) and 4.7 (95% CI, 4.3-5.0) among healthy and unhealthy WHR groups, respectively.

With adjustments of demographic, socioeconomic, lifestyle, and family history covariates (model 1), severe obesity, but not overweight or moderate obesity, was associated with higher mortality compared with normal weight (HR, 1.55; 95% CI, 1.08-2.22). The HRs comparing highest vs lowest body fat percentage groups (1.22; 95% CI, 0.92-1.64) and comparing unhealthy vs healthy WC groups (1.35; 95% CI, 0.98-1.85) were statistically nonsignificant, whereas the top WC quartile had an HR of 1.50 (95% CI, 1.01-2.24) compared with the bottom quartile (eTable 3 in Supplement 1). The HR comparing unhealthy and healthy WHR was 1.91 (95% CI, 1.28-2.86). With additional adjustments of baseline comorbidities (model 2), only WHR was associated with mortality (HR comparing unhealthy vs healthy WHR, 1.75; 95% CI, 1.17-2.62). After excluding participants with prevalent comorbidities, only WHR (HR comparing unhealthy vs healthy WHR, 2.15; 95% CI, 1.35-3.43) and WC (HR comparing unhealthy vs healthy WC, 1.84; 95% CI, 1.13-2.98) were associated with higher mortality (eTable 4 in Supplement 1). All associations were strengthened among never smokers. Results remained largely consistent in other sensitivity analyses (eTable 4 in Supplement 1).

### Subgroup Analyses

**Figure 2.**
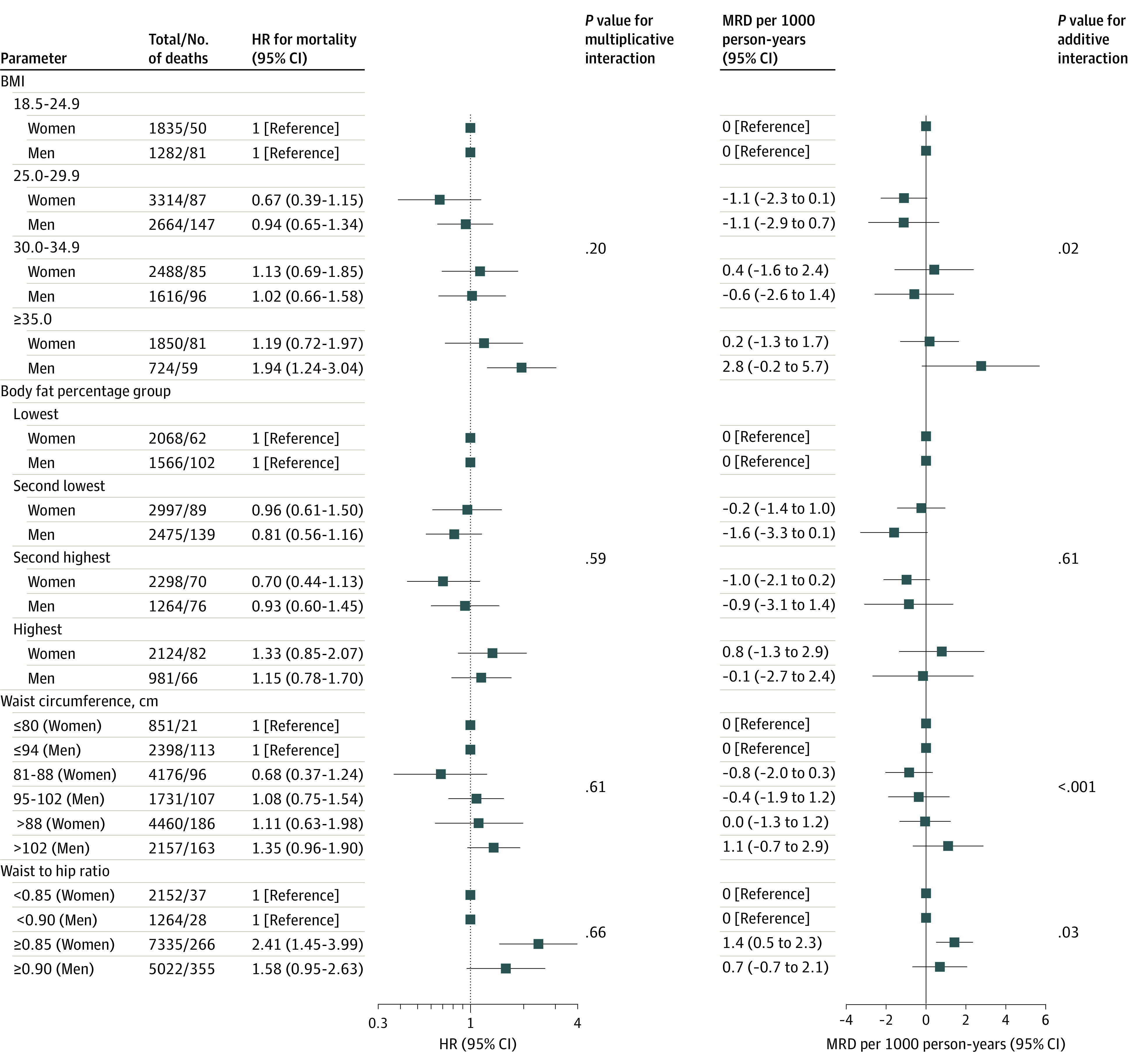
Associations Between Different Obesity Parameters and Mortality by Sex Data were adjusted for age, sex, field center, Hispanic or Latino background, marital status, income, educational level, employment status, insurance, length of stay in the US, preferred language, family history of myocardial infarction and diabetes, cigarette smoking, alcohol drinking, sleeping time, diet, and physical activity. BMI indicates body mass index (calculated as weight in kilograms divided by height in meters squared); HR, hazard ratio; and MRD, mortality rate difference.

Although no multiplicative interaction was detected, additive interactions were detected between sex and BMI, WC, and WHR (*P* < .03 for interaction) (Figure 2), indicating different MRDs by sex. The MRD between unhealthy and healthy WHR was 1.4 (95% CI, 0.5-2.3) per 1000 person-years among women and larger than 0.7 (95% CI, −0.7 to 2.1) among men. Restricted cubic splines showed that any increased WHR was associated with increased mortality among women, whereas higher WHR was associated only with higher mortality among men when WHR exceeded 1.00 (eFigure 3 in Supplement 1). Women with unhealthy WHR had greater WC but similar hip circumference compared with those with healthy WHR, whereas men with unhealthy WHR had both greater WC and hip circumference compared with those with healthy WHR (eTable 5 in Supplement 1).

In contrast, MRD between severe obesity and normal weight was 2.8 (95% CI, −0.2 to 5.7) per 1000 person-years among men, and larger than 0.2 (95% CI, −1.3 to 1.7) among women. Restricted cubic splines showed that higher BMI was associated with higher mortality when exceeding 32.4 and 42.2 among men and women, respectively (eFigure 3 in Supplement 1). Similar patterns were observed for WC. Associations of obesity parameters with mortality were largely consistent across other subgroups (eFigure 4 in Supplement 1).

### Independent, Interaction, and Joint Associations of General and Central Obesity With Mortality

**Figure 3.**
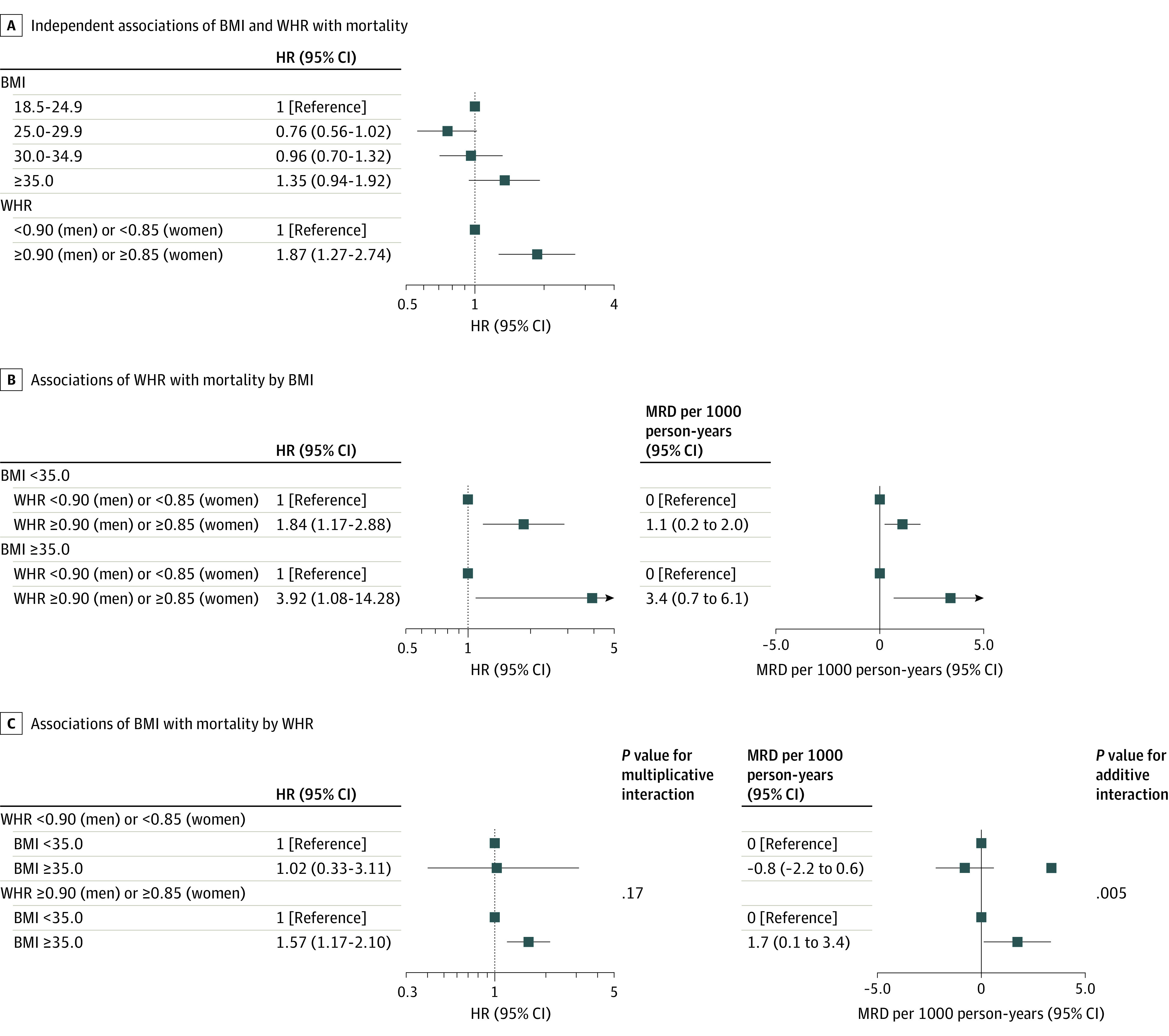
Independent Associations of Body Mass Index (BMI) and Waist to Hip Ratio (WHR) With Mortality Analyses considered complex survey design, including stratification, clustering, and sampling weights. Data were adjusted for age, sex, field center, Hispanic or Latino background, marital status, income, educational level, employment status, insurance, length of stay in the US, preferred language, family history of myocardial infarction and diabetes, cigarette smoking, alcohol drinking, sleeping time, diet, and physical activity. The BMI values were calculated as weight in kilograms divided by height in meters squared. HR indicates hazard ratio; MRD, mortality rate difference.

**Figure 4.**
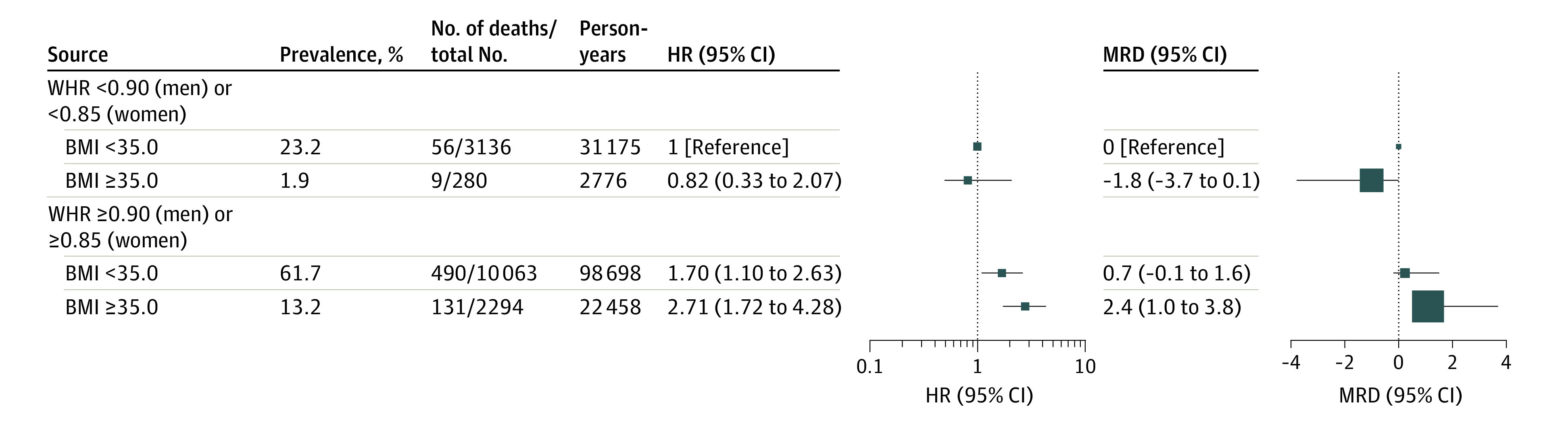
Joint Associations of Body Mass Index (BMI) and Waist to Hip Ratio (WHR) With Mortality The reference group is adults with a BMI of less than 35.0 (calculated as weight in kilograms divided by height in meters squared) and WHR less than 0.90 (men) or 0.85 (women). Analyses considered complex survey design, including stratification, clustering, and sampling weights. Data were adjusted for age, sex, field center, Hispanic or Latino background, marital status, income, educational level, employment status, insurance, length of stay in the US, preferred language, family history of myocardial infarction and diabetes, cigarette smoking, alcohol drinking, sleeping time, diet, and physical activity.

With mutual adjustment of BMI and WHR (Figure 3), the HRs were 1.87 (95% CI, 1.27-2.74) comparing unhealthy vs healthy WHR and 1.35 (95% CI, 0.94-1.92) comparing severe obesity vs normal weight. Interaction was observed between BMI and WHR (*P* values for multiplicative and additive interactions were .17 and .005, respectively). Compared with a BMI less than 35.0, a BMI of 35.0 or greater was only associated with higher mortality among those with an unhealthy WHR (HR, 1.57; 95% CI, 1.17-2.10) rather than those with a healthy WHR (HR, 1.02; 95% CI, 0.33-3.11). The HR comparing unhealthy vs healthy WHR was larger among those with a BMI of 35.0 or greater (HR, 3.92; 95% CI, 1.08-14.28) than those with a BMI less than 35.0 (HR, 1.84; 95% CI, 1.17-2.88). Compared with adults with a BMI less than 35.0 and a WHR less than 0.90/0.85 (Figure 4), those with a BMI of 35.0 or greater but a WHR less than 0.90/0.85 had no significantly different mortality risk (HR, 0.82; 95% CI, 0.33-2.07; MRD, −1.8 per 1000 person-years; 95% CI, −3.7 to 0.1), whereas those with a BMI less than 35.0 but a WHR of 0.90/0.85 or greater (HR, 1.70; 95% CI, 1.10 to 2.63; MRD, 0.7; 95% CI, −0.1 to 1.6) and those with both a BMI of 35.0 or greater and a WHR of 0.90/0.85 or greater had higher absolute and relative mortality rates (HR, 2.71; 95% CI, 1.72-4.28; MRD, 2.4; 95% CI, 1.0-3.8).

Adults with a BMI of 35.0 or greater but a WHR less than 0.90/0.85 (1.9% of the population) had the highest mean hip circumference (131.5 cm) compared with other groups (102.6-124.3 cm) (eTable 6 in Supplement 1) and exhibited more favorable metabolic characteristics, such as lower fasting blood glucose, glycosylated hemoglobin, and triglyceride levels and higher high-density lipoprotein cholesterol levels compared with those with a WHR or 0.90/0.85 or greater irrespective of BMI. Without considering WHR, 13.8% of the population would be misclassified as having overweight or obesity based on BMI, and 10.4% would be misclassified as having normal weight (eTable 7 in Supplement 1).

## Discussion

Despite the well-established associations of overweight and general and central obesity with increased mortality risks among White and Asian populations,^5,6,7,20,21^ evidence from Hispanic and Latino populations remains scarce and needed, considering the unique physiologic, sociocultural, and lifestyle features of the US Hispanics and Latinos.^8,9,10,11^ Limited studies from Hispanic and Latino populations (mostly of Mexican heritage) reported that overweight was unassociated or associated with lower mortality^12,13,14,15,16,17,18^; however, the associations between obesity and mortality were inconsistent.^12,13,14,15,16,17,18^ Two studies did not separate moderate and severe obesity and reported that obesity was associated with lower mortality among elderly individuals but not among younger adults,^13,17^ suggesting possible confounding from severe diseases that contributed to unintentional weight loss and increased mortality risks. Two studies divided obesity into more groups and found that moderate obesity was not associated or was slightly associated with higher mortality, whereas severe obesity was associated with increased mortality.^14,16^ For example, the Mexico City Prospective Study reported that BMIs of 30.0 to 34.9, 35.0 to 39.9, and 40.0 or greater were associated with a 1.16-, 1.62-, and 2.10-fold risk of mortality, respectively,^14^ largely consistent with our findings. However, the participants were Mexican, and the evidence cannot be directly extrapolated to US Hispanic and Latino populations with more diverse backgrounds and cultures. Only the US National Health and Nutrition Examination Survey (1988-2004) investigated the association between body fat percentage and mortality among the Mexican American population and found no linear association.^15^

As for central obesity, 3 studies examined the association of WC with mortality among Hispanic and Latino populations, and all found positive associations.^12,14,15^ However, these studies had limitations, such as not adjusting for acculturation or Hispanic or Latino backgrounds and not considering different body fat distributions between sexes. With more adequate control for confounding and a population with more diverse backgrounds, our study reported that the top WC quartile was associated with higher mortality risks, but the association was attenuated and statistically nonsignificant after adjusting for baseline comorbidities. Additionally, WHR reflects both subcutaneous and abdominal adipose tissue,^21^ and only 1 prior study reported the association between greater WHR and higher mortality among Hispanics and Latinos,^14^ which was consistent with our study. Above all, WHR was independently associated with mortality regardless of BMI or comorbidities; thus, it might be a better indicator of mortality compared with BMI, body fat percentage, and WC among Hispanic and Latino populations and should be routinely examined and prioritized in health practice.

Moreover, we found that US Hispanic or Latino adults with BMIs of 35.0 or greater but WHRs less than 0.90/0.85 may represent a unique subpopulation with distinct body fat distribution, whose mortality risks were similar to those with both BMIs less than 35.0 and WHRs less than 0.90/0.85. Specifically, these individuals had the greatest hip circumference among all groups, indicating more fat accumulation in the gluteofemoral region. Previous research has linked gluteofemoral adipose tissue to lower rates of lipolysis, inflammation, and metabolic risks,^34^ and we found that adults with BMIs of 35.0 or greater but WHRs less than 0.90/0.85 had better glucose and lipid profiles compared with those with WHRs of 0.90/0.85 or greater regardless of their BMI levels. However, these findings need validation and mechanistic support.

We found that the MRD between the WHR groups was greater among women compared with men, which was consistent with a previous meta-analysis.^7^ Similar findings also exist in the association of WHR with risks of cardiometabolic diseases,^35,36^ which could be explained by the fact that women had greater increased metabolic risks related to visceral adipose tissue compared with men.^37,38^ Additionally, women with WHRs of 0.85 or greater had greater WC but similar hip circumference compared with women with WHRs less than 0.85 in the HCHS/SOL, indicating that increased WHR was mostly caused by increased WC; however, men with WHRs of 0.90 or greater had both greater WC and hip circumference than men with WHRs less than 0.90, and harms of greater WHR might be partly counteracted by increased gluteofemoral adipose tissue and reduced metabolic risks.^34^

On the other hand, we found that the MRD for BMI was stronger among men, consistent with a previous meta-analysis.^6^ Similar findings also exist in risks of cardiovascular disease,^39,40,41^ diabetes,^42^ and some cancers.^43^ These findings might be explained by the fact that, compared with their female counterparts, men with obesity have more abdominal and visceral fat accumulation,^44^ lower concentration of antiatherogenic and anti-inflammatory adiponectin,^45,46,47,48^ and higher cancer risks associated with higher concentrations of free insulin–like growth factor 1.^49,50^

In a US Hispanic and Latino population with diverse backgrounds, this study strived to control for confounding from acculturation, Hispanic or Latino background, dietary habits, and physical activity and evaluated associations of 4 general and central obesity parameters with mortality, as well as the interaction of general and central obesity. Reverse causation related to deaths occurring in the first few years of follow-up, comorbidities, and cigarette smoking were considered.

### Limitations

This study has several limitations. First, insufficient statistical power could result in nonsignificant results for moderate obesity and body fat percentage given the relatively low mortality rate. Results from interaction and subgroup analyses might be particularly limited by insufficient statistical power, which needs future validation. Second, although we censored deaths occurring in the first 2 years of follow-up and controlled for baseline comorbidities, reverse causation is still possible given a median of 10.0 years of follow-up and other uncontrolled severe diseases. In addition, the association between obesity and mortality could change with longer follow-ups. Third, body fat percentage was measured by bioelectrical impedance analysis, which is not the standard measurement and might underestimate body fat percentage.^25^ Fourth, death information was collected by both health records and proxy reports, possibly causing misclassification, and causes of death are not yet available in the HCHS/SOL.

## Conclusions

In this study of US Hispanic and Latino adults, WHR was independently associated with mortality regardless of baseline BMI and comorbidities, and severe obesity was associated only with higher mortality among adults with unhealthy WHR. Thus, prioritizing clinical screening, intervention, and health education for WHR can be an important public health strategy among US Hispanic and Latino adults. Sex differences were observed, suggesting the need for sex-specific strategies in obesity prevention and intervention. The interaction between general and central obesity needs validation from future studies with larger sample sizes. Future studies with longer follow-up duration and high-quality measurements of body fat are still warranted.

## Notes

Supplement 1. **eMethods.** Supplemental Methods **eFigure 1.** Flow Chart of the Study **eFigure 2.** Correlation Between Different Obesity Indicators **eFigure 3.** Nonlinear Dose-Response Associations Between Different Obesity Parameters and Mortality **eFigure 4.** Associations Between Different Obesity Parameters and Mortality: Subgroup Analyses **eTable 1.** Corrected Body Fat Percentage Corresponding to Body Mass Index Cutoffs by Sex, Age, and Hispanic/Latino Background **eTable 2.** Baseline Characteristics of Study Population by Body Mass Index Groups **eTable 3.** Associations Between Different Obesity Parameters and Mortality **eTable 4.** Hazard Ratios (95% Confidence Intervals) Related to Different Obesity Parameters for Mortality: Sensitivity Analyses **eTable 5.** Distribution of Risk Factors by Sex and Obesity Parameters **eTable 6.** Distribution of Risk Factors by Body Mass Index and Waist-to-Hip Ratio **eTable 7.** Prevalence of Groups With Different Body Mass Index and Waist-to-Hip Ratio

Supplement 2.
Data Sharing Statement
