# Supplementary material for: General or Central Obesity and Mortality Among US Hispanic and Latino Adults
Source: JAMA Netw Open. 2024 Jan 16;7(1):e2351070. doi: 10.1001/jamanetworkopen.2023.51070 (PMC10792478; doi:10.1001/jamanetworkopen.2023.51070)
Supplement: Supplement 1. — eMethods. Supplemental Methods eFigure 1. Flow Chart of the Study eFigure 2. Correlation Between Different Obesity Indicators eFigure 3. Nonlinear Dose-Response Associations Between Different Obesity Parameters and Mortality eFigure 4. Associations Between Different Obesity Parameters and Mortality: Subgroup Analyses eTable 1. Corrected Body Fat Percentage Corresponding to Body Mass Index Cutoffs by Sex, Age, and Hispanic/Latino Background eTable 2. Baseline Characteristics of Study Population by Body Mass Index Groups eTable 3. Associations Between Different Obesity Parameters and Mortality eTable 4. Hazard Ratios (95% Confidence Intervals) Related to Different Obesity Parameters for Mortality: Sensitivity Analyses eTable 5. Distribution of Risk Factors by Sex and Obesity Parameters eTable 6. Distribution of Risk Factors by Body Mass Index and Waist-to-Hip Ratio eTable 7. Prevalence of Groups With Different Body Mass Index and Waist-to-Hip Ratio [file jamanetwopen-e2351070-s001.pdf]

Supplementary Online Content

Zhang Y, Chen G-C, Stores-Alvarez D, et al. General or central obesity and mortality among US Hispanic and Latino adults. *JAMA Netw Open*. 2024;7(1):e2351070. doi:10.1001/jamanetworkopen.2023.51070

eMethods. Supplemental Methods
eFigure 1. Flow Chart of the Study
eFigure 2. Correlation Between Different Obesity Indicators
eFigure 3. Nonlinear Dose-Response Associations Between Different Obesity Parameters and Mortality
eFigure 4. Associations Between Different Obesity Parameters and Mortality: Subgroup Analyses
eTable 1. Corrected Body Fat Percentage Corresponding to Body Mass Index Cutoffs by Sex, Age, and Hispanic/Latino Background
eTable 2. Baseline Characteristics of Study Population by Body Mass Index Groups
eTable 3. Associations between Different Obesity Parameters and Mortality
eTable 4. Hazard Ratios (95% Confidence Intervals) Related to Different Obesity Parameters for Mortality: Sensitivity Analyses
eTable 5. Distribution of Risk Factors by Sex and Obesity Parameters
eTable 6. Distribution of Risk Factors by Body Mass Index and Waist-to-Hip Ratio
eTable 7. Prevalence of Groups with Different Body Mass Index and Waist-to-Hip Ratio

This supplementary material has been provided by the authors to give readers additional information about their work.

eMethods. Supplemental Methods

Study Population

The HCHS/SOL is an ongoing population-based cohort study. At baseline (2008-2011), four field centers from four metropolitan areas in the US – Chicago (Illinois), Miami (Florida), Bronx (New York), and San Diego (California) planned to recruit up to 4000 Hispanic/Latino adults (~1500 adults aged 18-44 and ~2500 adults aged 45-74) via a multi-stage probability sampling method. Finally, 16,415 Hispanic/Latino adults aged 18-74 years were recruited. Stable communities were selected so that participants could be contacted and examined over time. The following individuals will be excluded: (1) those planning to move away in the next 3 years, (2) those with severe health problems, disabilities, or mental problems and unable to complete informed consent and study. Participants could choose to use English or Spanish, as the HCHS/SOL had both English and Spanish interviewers and questionnaires. Participants have diverse backgrounds including Central and South American, Cuban, Dominican, Mexican, and Puerto Rican.

Assessment of Obesity Parameters

Participants were asked to wear scrub suits or light clothing. BMI was calculated as body weight (kg) divided by squared height (m). Height (nearest cm) was measured using the wall-mounted Seca 222 stadiometer (Seca, Hamburg, Germany). Participants were asked to remove their hair ornaments from the top of the head. The back of the head, shoulder blades, buttocks, and heels contacted the vertical backboard. Weight (nearest 0.1 kg) and body fat percentage (BF%, nearest 0.1%) were measured using the Tanita Body Composition Analyzer TBF-300A (Tanita Corporation of America, Inc, Arlington Heights, IL, USA). BF% was estimated by the bioelectrical impedance method and corrected based on the 18O dilution method (corrected BF%=18.86+0.66*BF% for women; corrected BF%=12.75+0.73*BF% for men). BF% was categorized according to a previous HCHS/SOL study based on sex, age, and Hispanic/Latino background. In brief, sex-, age-, and Hispanic/Latino-background-specific linear regression models for BF% regressed on 1/BMI were fitted, which were used to predict BF% corresponding with WHO BMI cutoffs, and cutoffs for lowest (corresponding to normal weight), second lowest (overweight), second highest (moderate obesity), and highest (severe obesity) BF% groups are shown in **eTable 1**.

Waist circumference (WC) and hip circumference (both nearest cm) were measured using Gulick II anthropometric tape. The participants were asked to stand and hold their clothing above the waist. The pants and underclothing of the participant were slightly lowered. From behind and to the right of the participant, the staff palpated the hip area to locate the right ilium, drew a horizontal line just above the uppermost lateral border of the right ilium, and crossed the line to indicate the mid-axillary line of the body. Then, the staff stood on the participant's right side and placed the anthropometric tape around the trunk in a horizontal plane at the level marked on the right side of the trunk. The mirror on the wall was used to ensure the correct horizontal alignment of the anthropometric tape. The recorder (if available) ensured that the tape was parallel to the floor, snug, and not compressing the skin. Measurements were made at the end of a normal expiration. The hip circumference was measured at the level of maximal protrusion of the gluteal muscles, which was verified by passing the tape above and below the observed maximum. The anthropometric tape would be kept horizontally at this level, snug, but not compressing the skin, and the measurement should be made from the participant's right side. The mirror on the wall was used to ensure the correct horizontal alignment of the anthropometric tape. The recorder (if available) verified the position of the tape from both the front and back. WHR was calculated as WC divided by hip circumference.

Assessment of Covariates

All covariate information was self-reported and collected via questionnaires. Hispanic/Latino backgrounds included Central American, Cuban, Dominican, Mexican, Puerto Rican, South American, and more than one/others. Marital status was grouped into single, married/living with a partner, and separated/divorced/widow(er). Annual household income was grouped into less than $30,000 and $30,000 or more. Education was evaluated according to total years of schooling and highest grade/level of achieved education, which was categorized into no high school diploma/General Educational Development (GED; ≤10 years of education), at most a high school diploma/GED (11-12 years of education), and greater than high school/GED (≥13 years of education; highest level of trade/vocational school, university/college; or trade school certificate, associate degree, bachelor's degree, etc.). employment status was categorized into retired and not currently employed, not retired and not currently employed, employed part-time (≤35 hours/week), and employed full-time (>35 hours/week). Health insurance coverage was categorized into no and yes, and insurance provided through employers or labor unions, individual plan, Medicaid, Medicare, military insurance, Indian Health Services, etc. were considered. Participants were asked about their places of birth and years lived in the US, and accordingly, participants were categorized into living in the US 50 states/DC for less than 10 years, 10 years or more, and born in the US. Language preference was categorized into Spanish and English. Cigarette smoking status, exposure

© 2023 Zhang Y al. *JAMA Network Open.*

years, and the daily number of used cigarettes were asked. For current smokers, cigarette pack-years were calculated as the average daily number of used cigarettes multiplied by smoking years divided by 20 (number of cigarettes per pack), and the median of cigarette pack years was 6.30. Participants were categorized into never smoking, former smoking, current light smoking (current smoking and below the median of cigarette pack years), and current heavy smoking (current smoking and above the median of cigarette pack years). Participants were asked about their current alcohol drinking status and total weekly alcohol consumption, and alcohol use was categorized into never drinking, former drinking, current low to moderate drinking (<7/14 drinks/wk for women/men), and current heavy drinking (≥7/14 drinks/wk for women/men). Sleep duration was calculated according to weekday and weekend sleep duration and categorized into 6-9 h/d and <6/>9 h/d. Healthy Eating Index 2010 was calculated according to consumption of fruit, juice, vegetables, greens and beans, whole grains, dairy, total protein foods, seafood and plant protein, fatty acids, refined grains, sodium, and total energy, which was categorized into quintiles. Global physical activity questionnaire was used for physical activity measurements, and total physical activity (weekly moderate/vigorous physical activity) was classified into high activity (>300/150 min), medium activity (150-300/75-150 min), low activity (0-149/0-74 min), and inactive (no activity). Family history of myocardial infarction and diabetes, and prevalent diabetes, cardiovascular disease, cancer except for nonmelanoma skin cancer, and chronic obstructive pulmonary disease were self-reported by participants. Additionally, if participants used glucose-lowering medications or had fasting blood glucose level of ≥126 mg/dL, blood glucose level of 2-hour post-oral glucose tolerance test of ≥200 mg/dl, or glycosylated hemoglobin level of ≥6.5%, they would also be defined as diabetes cases. Cardiovascular disease included prevalent heart disease (i.e., self-reported physician-diagnosed heart attack, self-reported procedures of a balloon angioplasty, a stent, or bypass surgery, or electrocardiogram reports of major Q wave abnormalities or minor Q/QS waves with ST/T abnormalities) and cerebrovascular disease or carotid revascularization (i.e., self-reported stroke, mini-stroke or transient ischemic attack, or procedures of a balloon angioplasty or surgery to the arteries of necks to prevent or correct a stroke). Emphysema and chronic bronchitis were also considered as chronic obstructive pulmonary disease.

Subgroup Analyses

Subgroup analyses by sex, age, Hispanic/Latino background, acculturation, socioeconomic status, cigarette smoking, physical activity, and dietary quality were conducted. Multiplicative and additive interactions were evaluated by introducing a product term of the obesity parameter and subgroup variable into Cox proportional hazard regression and additive hazards model, and we focused on the interactions of the highest vs lowest categories of obesity parameters, which was of public health interest.

Each subgroup variable was categorized into two levels. Sex was grouped into men and women. Age was grouped into <60 years and ≥60 years. Three major Hispanic/Latino backgrounds were considered, i.e., Cuban (yes and no), Mexican (yes and no), and Puerto Rican (yes and no).

Acculturation was evaluated by an acculturation score according to the Multi-Ethnic Study of Atherosclerosis. A 5-point acculturation score was calculated according to nativity, years in the US, and language preference, with a higher score indicating more accultured. Nativity and years in the U.S. were combined to assign a score of 3 for US-born participants, 2 for foreign-born participants who lived in the US for over 20 years, 1 for foreign-born participants who lived in the US for 10-19 years, and 0 for foreign-born participants who lived in the US for less than 10 years. Preferred language was used to assign a score of 2 for preference to English, 1 for equal preference to English and Spanish, and 0 for preference to Spanish. The acculturation score was obtained by summing these scores, and participants were grouped into more accultured (scores of 3-5) and less accultured (scores of 0-2) according to the midpoint of the score range.

A socioeconomic score was constructed according to income, education, and employment. Each socioeconomic factor was categorized into two groups; the advantaged group (i.e., yearly household income ≥$30,000, education level of high school or higher, and being employed) received one point, and the disadvantaged group received zero points. The sum of the points was the socioeconomic score, and participants were grouped into low (0-1 point) and high socioeconomic groups (2-3 points).

Physical activity was grouped into high activity (weekly moderate/vigorous physical activity >300/150 min) and no high activity (≤300/150 min). Dietary quality was grouped into above and below the median of the Healthy Eating Index 2010.

More Stringent Criteria to Ascertain Death

In the main analysis, deaths were determined via death certificates, the National Death Index, and proxy reports during annual telephone follow-ups. All death records from death certificates and the National Death Index provided specific dates of death, which have been updated until December 31, 2019. Most proxy reports during annual telephone follow-ups provided specific dates of death, while dates of death were unavailable for 104 out of 686 deaths, and the date of the telephone follow-up will be used as the date of death.

In the sensitivity analysis, we used more stringent criteria to ascertain deaths. This definition only included deaths

© 2023 Zhang Y al. *JAMA Network Open.*

identified via death certificates, the National Death Index, and proxy reports during annual telephone follow-ups that reported dates of death. Additionally, follow-up time started from baseline until the date of death, loss to follow-up, or December 31, 2019, whichever occurred first.

© 2023 Zhang Y al. *JAMA Network Open.*

eFigure 1. Flow Chart of the Study

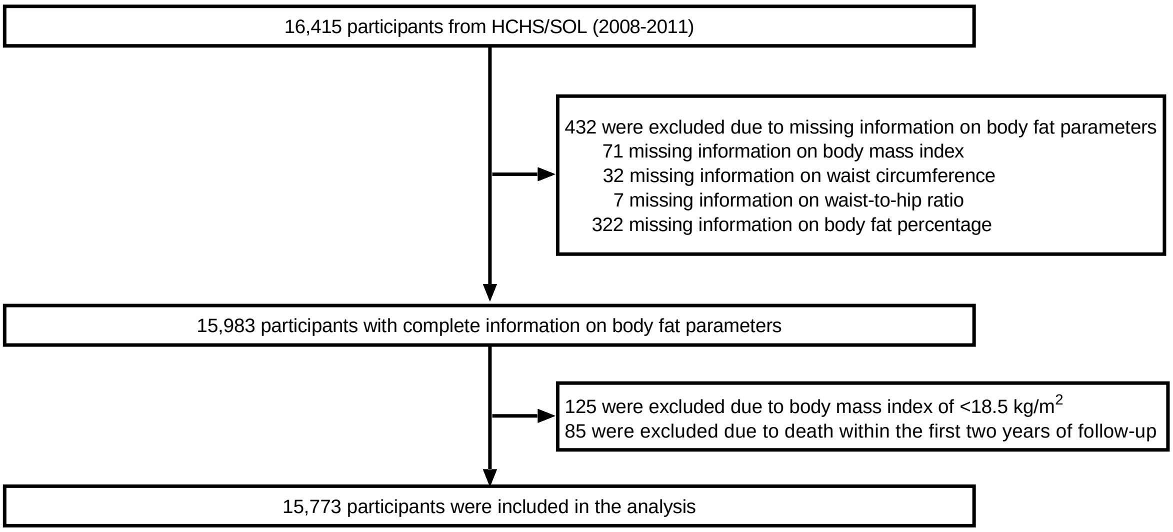

We used subpopulation or domain statements to restrict the analyses to the eligible participants, and those “excluded” participants also contributed to the variance calculations. HCHS/SOL indicates Hispanic Community Health Study/Study of Latinos.

© 2023 Zhang Y al. *JAMA Network Open.*

eFigure 2. Correlation between Different Obesity Indicators

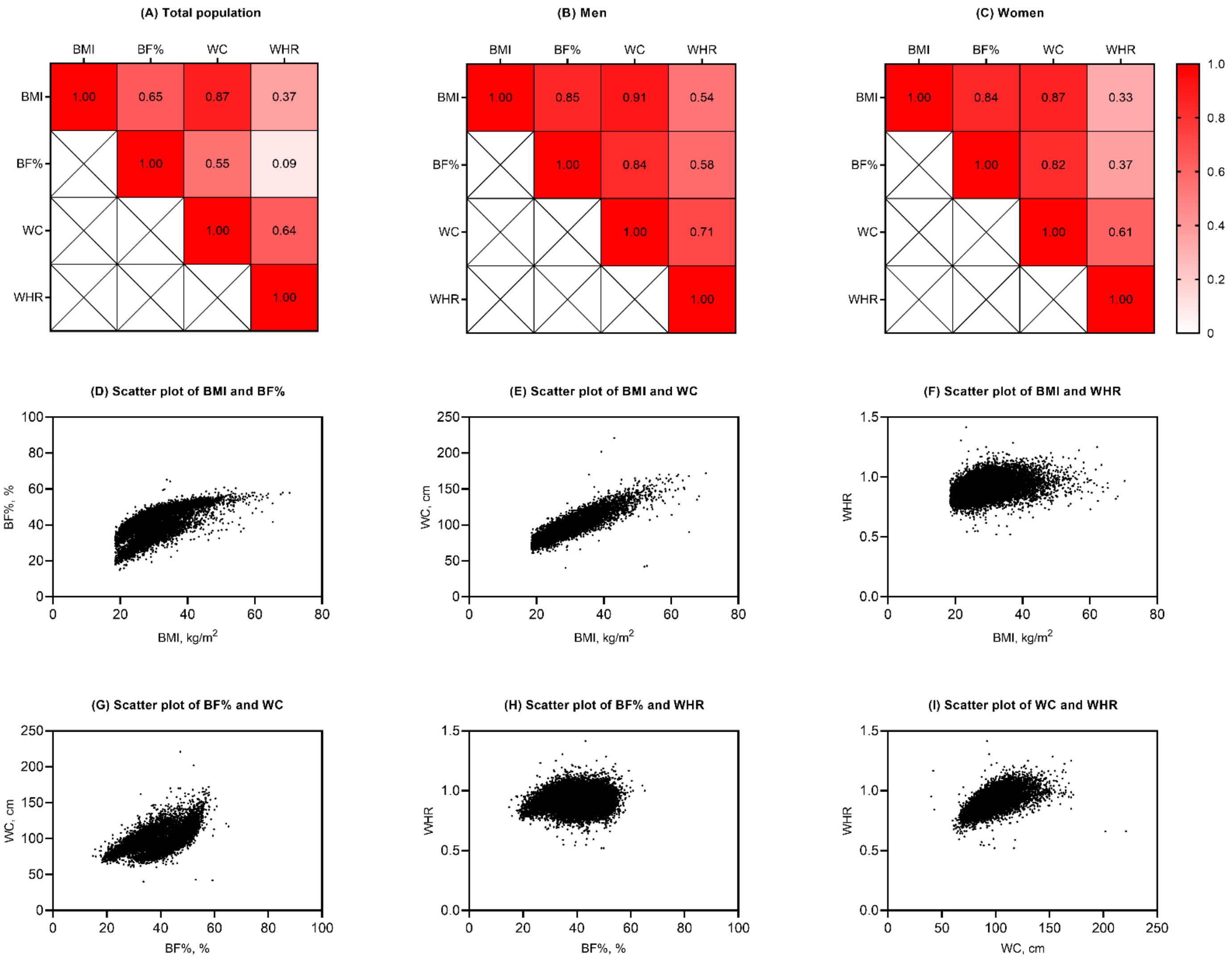

All *P* values for correlations were <.001. BF% indicates body fat percentage; BMI, body mass index; WC, waist circumference; WHR, waist-to-hip ratio.

© 2023 Zhang Y al. *JAMA Network Open.*

eFigure 3. Nonlinear Dose-Response Associations between Different Obesity Parameters and Mortality

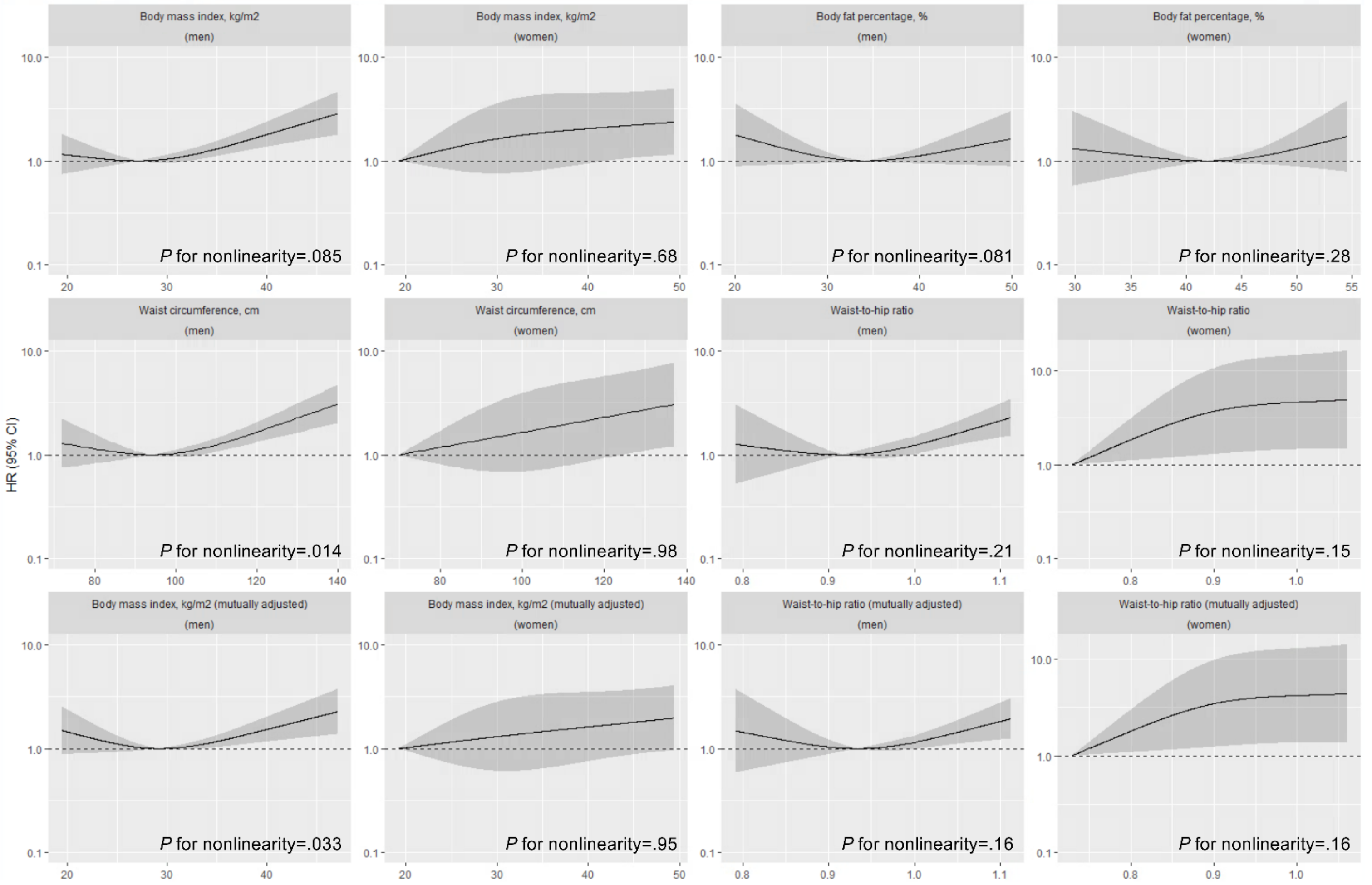

Restricted cubic splines were used to investigate the possible nonlinear dose-response association, and Hispanic Community Health Study/Study of Latinos complex survey design, including stratification, clustering, and sampling weights were considered. Three knots at the 5th, 50th, and 95th percentiles of the distributions of different obesity parameters were used. Age, sex, field center, Hispanic/Latino background, marital status, income, education, employment status, insurance, length of staying in the US, preferred language, family history of myocardial infarction and diabetes, cigarette smoking, alcohol drinking, sleeping time, diet, and physical activity were controlled. The bottom row shows the results that body mass index and waist-to-hip ratio are mutually adjusted. Reference levels were set as the values for the lowest risk.

© 2023 Zhang Y al. *JAMA Network Open.*

eFigure 4. Associations between Different Obesity Parameters and Mortality: Subgroup Analyses

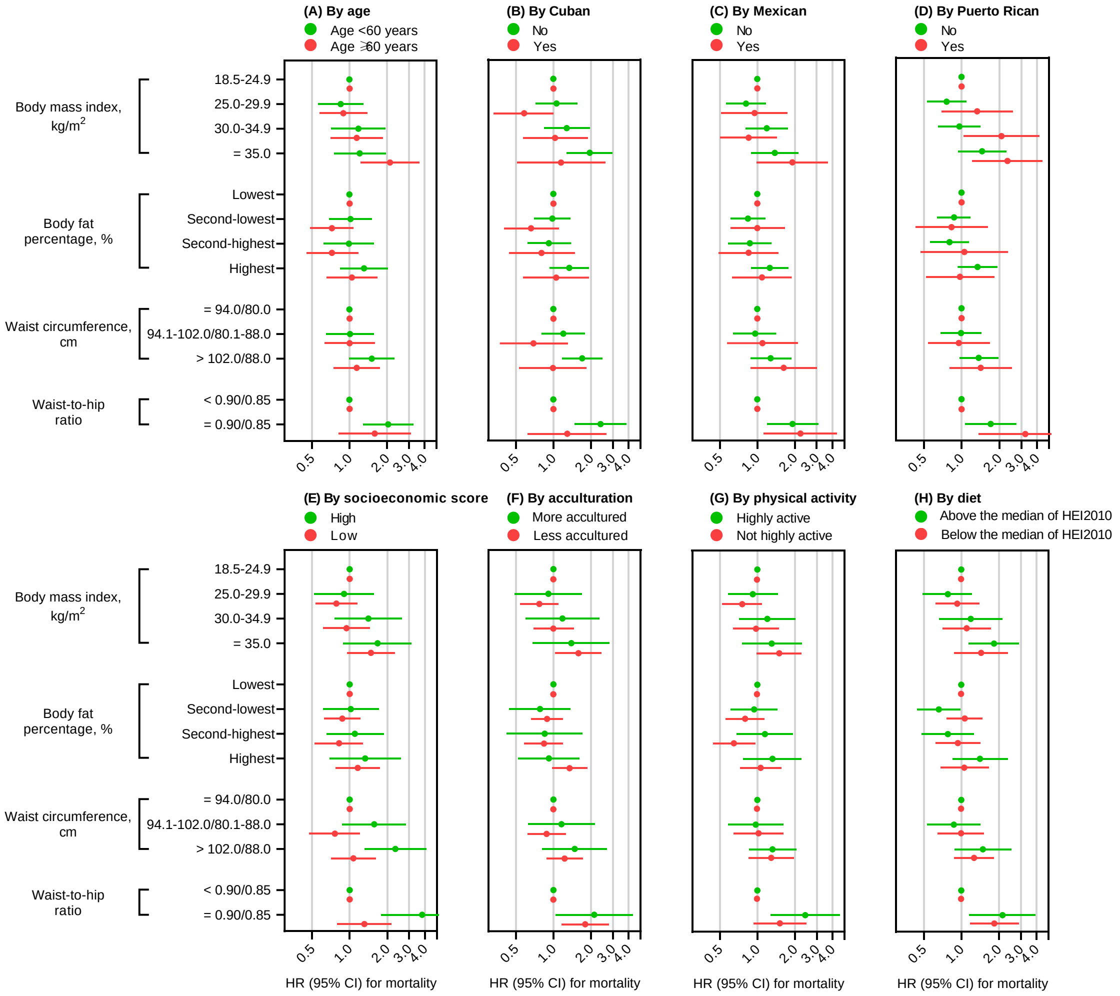

Analyses considered complex survey design, including stratification, clustering, and sampling weights. Age, sex, field center, Hispanic background, marital status, income, education, employment status, insurance, length of staying in the US, preferred language, family history of myocardial infarction and diabetes, cigarette smoking, alcohol drinking, sleeping time, diet, and physical activity. All *P* for multiplicative interaction>.05, except the association of waist-to-hip ratio between socioeconomic score (*P*=.031). CI indicates confidence interval; HR, hazard ratio.

© 2023 Zhang Y al. *JAMA Network Open.*

eTable 1. Corrected Body Fat Percentage Corresponding to Body Mass Index Cutoffs by Sex, Age, and Hispanic/Latino Background[a]

Sex	Age, years	Hispanic/Latino background	Body mass index, kg/m^2		
			25	30	35
Women	18-29	Mexican	39.8	44.6	48.0
Women	18-29	Dominican	40.2	44.7	47.9
Women	18-29	Central American	39.5	44.2	47.5
Women	18-29	Cuban	39.9	44.6	47.9
Women	18-29	Puerto Rican	39.9	44.6	47.9
Women	18-29	South American	39.5	44.3	47.8
Women	18-29	Other	39.8	44.6	47.9
Women	30-49	Mexican	40.6	44.8	47.8
Women	30-49	Dominican	41.1	45.2	48.2
Women	30-49	Central American	40.3	44.7	47.9
Women	30-49	Cuban	41.1	45.4	48.5
Women	30-49	Puerto Rican	40.8	44.9	47.8
Women	30-49	South American	40.4	44.7	47.8
Women	30-49	Other	40.7	45.0	48.0
Women	50-76	Mexican	41.9	45.9	48.8
Women	50-76	Dominican	41.9	45.9	48.7
Women	50-76	Central American	41.4	45.7	48.8
Women	50-76	Cuban	42.6	46.6	49.4
Women	50-76	Puerto Rican	41.4	45.5	48.4
Women	50-76	South American	41.7	45.8	48.8
Women	50-76	Other	42.0	46.0	48.8
Men	18-29	Mexican	28.0	33.8	38.0
Men	18-29	Dominican	28.6	34.3	38.4
Men	18-29	Central American	28.3	33.4	37.0
Men	18-29	Cuban	28.3	33.7	37.6
Men	18-29	Puerto Rican	27.6	33.5	37.7
Men	18-29	South American	27.5	32.4	35.9
Men	18-29	Other	28.1	33.8	37.8
Men	30-49	Mexican	28.8	34.8	39.1
Men	30-49	Dominican	30.1	35.0	38.6
Men	30-49	Central American	29.3	34.7	38.6
Men	30-49	Cuban	29.5	34.9	38.8
Men	30-49	Puerto Rican	29.2	34.5	38.3
Men	30-49	South American	28.8	34.4	38.3
Men	30-49	Other	29.2	34.8	38.8
Men	50-76	Mexican	30.7	35.6	39.1
Men	50-76	Dominican	30.8	35.7	39.2
Men	50-76	Central American	30.3	35.6	39.4
Men	50-76	Cuban	30.8	35.7	39.3
Men	50-76	Puerto Rican	30.5	35.4	38.8
Men	50-76	South American	30.1	35.1	38.7
Men	50-76	Other	30.7	35.6	39.1

[a] Values are corrected body fat percentage corresponding to the body mass index from a previous Hispanic Community Health Study/Study of Latinos.

© 2023 Zhang Y al. *JAMA Network Open.*

eTable 2. Baseline Characteristics of Study Population by Body Mass Index Groups[a]

Variable	Body mass index (kg/m²) 18.5-24.9	25.0-29.9	30.0-34.9	≥35.0
n (unweighted)	3117	5978	4104	2574
Age, years	35.8 (0.4)	42.5 (0.3)	43.0 (0.4)	41.4 (0.5)
Body mass index, kg/m²	22.8 (0.0)	27.5 (0.0)	32.2 (0.0)	39.9 (0.1)
Waist circumference, cm	83.6 (0.2)	93.9 (0.1)	104.2 (0.2)	118.7 (0.4)
Waist-to-hip ratio	0.9 (0.0)	0.9 (0.0)	0.9 (0.0)	1.0 (0.0)
Body fat percentage, %	32.7 (0.2)	37.4 (0.1)	41.8 (0.2)	47.4 (0.2)
Men, %	47.3 (1.3)	51.8 (0.9)	48.6 (1.2)	35.1 (1.5)
Field center, %				
Bronx	23.5 (1.6)	26.8 (1.6)	28.8 (1.7)	32.8 (2.1)
Chicago	14.6 (1.2)	15.8 (1.1)	17.0 (1.2)	15.2 (1.2)
Miami	36.7 (2.5)	30.1 (2.2)	28.6 (2.1)	27.0 (2.5)
San Diego	25.2 (1.9)	27.3 (2.1)	25.5 (1.8)	25.0 (1.9)
Hispanic/Latino background, %				
Central American	7.4 (0.7)	7.7 (0.6)	7.5 (0.7)	6.2 (0.7)
Cuban	25.1 (2.0)	20.3 (1.7)	20.1 (1.7)	19.4 (1.9)
Dominican	8.3 (0.8)	9.7 (0.8)	10.2 (0.9)	9.8 (1.2)
Mexican	33.8 (1.8)	39.1 (2.0)	38.9 (1.7)	33.2 (1.9)
Puerto Rican	14.4 (1.0)	13.9 (0.9)	15.1 (1.0)	22.9 (1.7)
South American	6.6 (0.7)	5.6 (0.4)	4.2 (0.4)	3.1 (0.5)
More than one/others	4.4 (0.5)	3.7 (0.4)	4.1 (0.6)	5.4 (0.7)
Marital status, %				
Married or living with a partner	44.9 (1.5)	52.1 (1.0)	51.8 (1.2)	46.9 (1.5)
Separated, divorced, or widow(er)	20.5 (1.1)	17.4 (0.7)	16.9 (0.8)	20.1 (1.2)
Single	34.3 (1.0)	30.2 (0.9)	30.9 (1.1)	32.5 (1.2)
Yearly household income <$30,000, %	59.4 (1.4)	60.9 (1.3)	62.1 (1.3)	65.0 (1.6)
Education, %				
>High school/GED	43.0 (1.4)	38.7 (1.2)	38.5 (1.3)	36.9 (1.5)
High school graduate/GED	26.2 (1.0)	27.4 (0.9)	26.0 (1.1)	28.5 (1.3)
<High school/GED	30.4 (1.3)	33.6 (1.1)	35.1 (1.2)	34.3 (1.4)
Employment status, %				
Employed full-time	32.6 (1.1)	34.9 (0.9)	34.8 (1.1)	25.5 (1.3)
Employed part-time	17.8 (0.9)	16.4 (0.7)	14.8 (0.8)	15.0 (1.1)
Retired	9.1 (0.5)	9.7 (0.5)	9.7 (0.7)	12.3 (1.0)
Unemployed	38.8 (1.1)	37.1 (1.1)	38.7 (1.2)	45.4 (1.4)
No current health insurance, %	49.4 (1.4)	49.0 (1.2)	48.6 (1.4)	43.2 (1.5)
Nativity and residence in US, %				
US born	18.9 (1.0)	18.1 (0.9)	21.1 (1.1)	29.6 (1.3)
Non-US born, residence≥10 years	49.1 (1.2)	51.8 (1.0)	53.8 (1.3)	52.0 (1.2)
Non-US born, residence<10 years	31.2 (1.4)	29.7 (1.2)	24.6 (1.3)	17.7 (1.2)
Preferred to speak in Spanish, %	79.0 (1.1)	80.2 (0.9)	76.2 (1.3)	66.0 (1.9)
Cigarette smoking, %				
Never	59.3 (1.3)	61.6 (1.0)	58.9 (1.1)	60.7 (1.7)
Former	14.0 (0.9)	18.3 (0.8)	20.7 (0.9)	19.4 (1.2)
Current light	9.2 (0.7)	10.1 (0.6)	10.8 (0.8)	10.7 (1.5)
Current heavy	17.2 (1.1)	9.7 (0.6)	9.0 (0.7)	8.4 (0.8)

© 2023 Zhang Y al. *JAMA Network Open.*

Alcohol drinking, %				
Never	21.0 (1.2)	18.4 (0.8)	18.7 (1.1)	19.4 (1.3)
Former	26.6 (1.1)	28.9 (1.0)	30.8 (1.2)	35.1 (1.5)
Current moderate	46.4 (1.1)	46.6 (1.2)	43.6 (1.4)	39.8 (1.6)
Current heavy	5.8 (0.5)	5.9 (0.4)	6.6 (0.7)	5.4 (0.7)
Sleep <6 or >9 h/d	25.3 (1.1)	22.6 (0.8)	24.3 (1.1)	25.9 (1.4)
Family history of myocardial infarction, %	28.2 (1.1)	30.2 (0.8)	29.5 (1.1)	33.8 (1.4)
Family history of diabetes, %	33.0 (1.3)	40.6 (1.1)	43.2 (1.3)	50.7 (1.6)
Physical activity				
High activity	57.6 (1.2)	56.3 (0.9)	55.1 (1.2)	46.9 (1.5)
Medium activity	10.7 (0.7)	10.9 (0.6)	9.8 (0.7)	10.8 (0.9)
Low activity	11.4 (0.9)	12.6 (0.6)	12.5 (0.8)	14.2 (1.1)
No activity	19.6 (1.0)	19.6 (0.8)	21.6 (0.9)	27.4 (1.3)
Healthy eating index-2010, %				
Quintile 5	22.4 (1.2)	20.8 (0.9)	20.6 (1.1)	20.0 (1.2)
Quintile 4	19.4 (1.0)	21.0 (0.8)	19.9 (1.0)	18.6 (1.2)
Quintile 3	19.4 (1.0)	21.4 (0.7)	23.1 (1.1)	19.4 (1.1)
Quintile 2	20.0 (1.1)	18.8 (0.8)	19.3 (1.0)	20.3 (1.3)
Quintile 1	18.8 (1.0)	18.0 (0.8)	17.2 (0.9)	21.8 (1.3)
Prevalent diabetes, %	11.0 (0.7)	15.1 (0.7)	20.5 (0.8)	28.5 (1.2)
Prevalent cardiovascular disease, %	6.3 (0.6)	6.6 (0.4)	9.6 (0.8)	11.3 (1.0)
Prevalent cancer, %	3.0 (0.4)	3.4 (0.4)	3.8 (0.5)	4.7 (0.6)
Prevalent chronic obstructive pulmonary diseases, %	6.6 (0.7)	5.0 (0.4)	5.4 (0.5)	11.5 (1.2)

[a] The analysis considered complex and multistage probability sampling (i.e., sampling weight, stratification, and clustering), and all estimates were standardized to the United States 2010 Decennial Census age distribution. Data are shown as mean (standard error) for continuous variables and percentage (standard error) for categorical variables.

© 2023 Zhang Y al. *JAMA Network Open.*

eTable 3. Associations between Different Obesity Parameters and Mortality[a]

Indicators	Quartile 1	Quartile 2	Quartile 3	Quartile 4
Body mass index				
Model 1	Reference	0.76 (0.55-1.07)	0.89 (0.63-1.24)	1.35 (0.97-1.88)
Model 2	Reference	0.75 (0.54-1.04)	0.83 (0.59-1.17)	1.17 (0.85-1.61)
Body fat percentage				
Model 1	Reference	1.02 (0.75-1.40)	0.76 (0.56-1.05)	1.11 (0.82-1.52)
Model 2	Reference	0.99 (0.73-1.34)	0.75 (0.55-1.01)	0.96 (0.71-1.31)
Waist circumference				
Model 1	Reference	0.92 (0.61-1.37)	1.05 (0.72-1.55)	1.50 (1.01-2.24)
Model 2	Reference	0.86 (0.58-1.28)	1.00 (0.69-1.45)	1.25 (0.84-1.84)
Waist-to-hip ratio				
Model 1	Reference	1.63 (1.05-2.53)	1.64 (1.05-2.57)	2.19 (1.35-3.54)
Model 2	Reference	1.60 (1.03-2.49)	1.52 (0.96-2.40)	1.91 (1.18-3.10)

[a] Analyses considered complex survey design, including stratification, clustering, and sampling weights. Deaths occurring within the first two years of follow-up were censored to reduce the possibility of reverse causation. Model 1 adjusted for age, sex, field center, Hispanic/Latino background, marital status, income, education, employment status, insurance, length of staying in the US, preferred language, family history of myocardial infarction and diabetes, cigarette smoking, alcohol drinking, sleeping time, diet, and physical activity. Model 2 additionally adjusted for prevalent diabetes, cardiovascular disease, cancer, and chronic obstructive pulmonary disease.

© 2023 Zhang Y al. *JAMA Network Open.*

eTable 4. Hazard Ratios (95% Confidence Intervals) Related to Different Obesity Parameters for Mortality: Sensitivity Analyses[a]

							Analysis 7: excluding			
Indicators (men/women)[b]	Analysis 1	Analysis 2	Analysis 3	Analysis 4	Analysis 5	Analysis 6	Bronx	Chicago	Miami	San Diego
Body mass index, kg/m^2										
18.5-24.9	Reference	Reference	Reference	Reference	Reference	Reference	Reference	Reference	Reference	Reference
25.0-29.9	1.02 (0.67-1.56)	0.94 (0.71-1.25)	0.85 (0.61-1.18)	0.95 (0.56-1.62)	0.84 (0.62-1.15)	0.89 (0.66-1.21)	0.79 (0.55-1.12)	0.84 (0.60-1.18)	0.99 (0.66-1.49)	0.81 (0.58-1.13)
30.0-34.9	1.39 (0.82-2.35)	1.17 (0.86-1.60)	1.09 (0.76-1.56)	1.52 (0.85-2.72)	1.13 (0.81-1.59)	1.01 (0.72-1.42)	0.96 (0.66-1.41)	1.17 (0.81-1.69)	1.23 (0.78-1.93)	1.11 (0.76-1.62)
≥35.0	1.00 (0.54-1.88)	1.37 (0.99-1.89)	1.84 (1.25-2.69)	2.31 (1.38-3.85)	1.61 (1.13-2.28)	1.51 (1.07-2.13)	1.56 (1.02-2.41)	1.63 (1.10-2.42)	1.89 (1.20-2.97)	1.26 (0.84-1.89)
Body fat percentage, %										
Lowest	Reference	Reference	Reference	Reference	Reference	Reference	Reference	Reference	Reference	Reference
Second lowest	1.11 (0.76-1.62)	0.91 (0.71-1.15)	0.81 (0.59-1.12)	0.81 (0.51-1.29)	0.81 (0.61-1.08)	0.79 (0.60-1.03)	0.89 (0.62-1.30)	1.03 (0.71-1.48)	1.09 (0.72-1.65)	0.92 (0.64-1.32)
Second highest	1.36 (0.81-2.28)	0.91 (0.68-1.23)	0.94 (0.65-1.37)	0.88 (0.52-1.48)	0.85 (0.60-1.21)	0.75 (0.53-1.06)	1.28 (0.89-1.84)	1.45 (1.01-2.09)	1.51 (1.01-2.24)	1.22 (0.86-1.75)
Highest	1.26 (0.76-2.09)	1.11 (0.85-1.45)	1.20 (0.84-1.71)	1.71 (1.10-2.64)	1.21 (0.88-1.65)	1.09 (0.80-1.47)	1.00 (1.00-1.00)	1.00 (1.00-1.00)	1.00 (1.00-1.00)	1.00 (1.00-1.00)
Waist circumference, cm										
≤94.0/80.0	Reference	Reference	Reference	Reference	Reference	Reference	Reference	Reference	Reference	Reference
94.1-102.0/80.1-88.0	1.41 (0.94-2.11)	1.03 (0.75-1.39)	1.01 (0.69-1.48)	1.54 (0.85-2.79)	0.96 (0.69-1.34)	0.92 (0.67-1.27)	1.00 (1.00-1.00)	1.00 (1.00-1.00)	1.00 (1.00-1.00)	1.00 (1.00-1.00)
>102.0/88.0	1.84 (1.13-2.98)	1.46 (1.10-1.94)	1.35 (0.94-1.94)	2.15 (1.18-3.90)	1.31 (0.94-1.83)	1.18 (0.87-1.62)	0.88 (0.65-1.20)	0.87 (0.64-1.18)	0.87 (0.62-1.22)	0.83 (0.62-1.11)
Waist-to-hip ratio										
<0.90/0.85	Reference	Reference	Reference	Reference	Reference	Reference	Reference	Reference	Reference	Reference
≥0.90/0.85	2.15 (1.35-3.43)	2.41 (1.70-3.43)	1.73 (1.11-2.68)	2.94 (1.60-5.39)	1.76 (1.17-2.66)	1.72 (1.16-2.55)	1.16 (0.84-1.61)	1.30 (0.95-1.78)	1.24 (0.85-1.82)	1.14 (0.83-1.58)

[a] Analyses considered complex survey design, including stratification, clustering, and sampling weights. Age, sex, field center, Hispanic/Latino background, marital status, income, education, employment status, insurance, length of staying in the US, preferred language, family history of myocardial infarction and diabetes, cigarette smoking, alcohol drinking, sleeping time, diet, and physical activity were controlled. Analysis 1 excluded participants with prevalent diabetes, cardiovascular disease, cancer, and chronic obstructive pulmonary disease. Analysis 2 did not censor deaths occurring within the first two years of follow-up. Analysis 3 used a more stringent criteria to ascertain deaths. Analysis 4 only included never smokers. Analysis 5 used multiple imputations to impute missing covariates. Analysis 6 treated obesity parameters as time-varying variables by including the measurements at the first follow-up (2014-2017). Analysis 7 excluded participants from one field center at a time.

[b] Groups of body fat percentage are defined in **Supplementary Table 1**.

© 2023 Zhang Y al. *JAMA Network Open.*

eTable 5. Distribution of Risk Factors by Sex and Obesity Parameters[a]

	Women		Men		Women		Men	
	Body mass index, kg/m^2				Waist-to-hip ratio			
Risk factors	<25.0	≥35.0	<25.0	≥35.0	<0.85	≥0.85	<0.90	≥0.90
Waist-to-hip ratio	0.86 (0.00)	0.92 (0.00)	0.90 (0.00)	1.01 (0.00)	0.81 (0.00)	0.93 (0.00)	0.87 (0.00)	0.97 (0.00)
Body mass index, kg/m^2	22.8 (0.1)	40.3 (0.2)	22.9 (0.1)	39.3 (0.2)	28.2 (0.3)	30.9 (0.1)	25.2 (0.1)	30.3 (0.1)
Waist circumference, cm	82.4 (0.2)	116.5 (0.5)	84.8 (0.2)	123.1 (0.6)	87.0 (0.4)	100.9 (0.3)	86.6 (0.3)	102.8 (0.3)
Hip circumference, cm	95.7 (0.2)	126.8 (0.4)	94.1 (0.2)	122.0 (0.6)	108.0 (0.5)	108.8 (0.3)	100.0 (0.4)	105.6 (0.3)
Age, years	35.8 (0.5)	42.8 (0.7)	35.8 (0.6)	39.0 (0.7)	36.8 (0.5)	43.7 (0.4)	31.3 (0.4)	43.1 (0.3)
Single, %	29.5 (1.1)	31.7 (1.5)	40.1 (1.7)	32.6 (1.8)	29.9 (1.2)	29.6 (0.9)	42.0 (1.9)	31.2 (0.9)
Yearly household income <$30,000, %	59.9 (1.8)	68.8 (1.9)	58.7 (2.0)	58.9 (2.6)	62.4 (1.8)	65.7 (1.0)	58.0 (2.2)	58.7 (1.4)
<High school/GED, %	27.5 (1.5)	35.8 (1.8)	33.8 (1.8)	30.9 (2.1)	26.7 (1.7)	35.6 (1.0)	31.3 (1.8)	34.2 (1.1)
Unemployed, %	43.6 (1.4)	49.8 (1.8)	33.4 (1.8)	36.4 (2.1)	44.7 (1.7)	47.8 (1.0)	34.3 (2.3)	30.4 (1.0)
No current health insurance, %	48.2 (1.6)	40.4 (1.8)	51.2 (1.9)	47.1 (2.3)	44.7 (1.8)	46.2 (1.1)	47.2 (2.1)	50.7 (1.2)
Reside in US <10 years, %	33.1 (1.7)	19.8 (1.6)	28.8 (1.9)	13.9 (1.6)	32.5 (1.8)	26.2 (1.0)	29.2 (2.5)	24.2 (1.1)
Preferred to speak in Spanish, %	81.1 (1.3)	67.4 (2.5)	76.4 (1.7)	65.0 (2.0)	77.0 (2.0)	77.6 (1.1)	76.2 (1.8)	75.3 (1.1)
Cigarette pack years, pack-years[b]	3.7 (0.5)	4.1 (0.4)	11.2 (0.8)	8.7 (1.0)	2.4 (0.3)	4.0 (0.2)	10.3 (1.0)	7.8 (0.3)
Former or heavy alcohol drinking, %	32.0 (1.5)	41.1 (2.2)	32.6 (1.6)	39.6 (2.4)	34.9 (2.0)	37.4 (1.0)	33.0 (1.9)	37.1 (1.0)
Sleep of <6 or >9 h/d, %	25.4 (1.5)	26.8 (1.8)	25.7 (1.6)	23.6 (1.9)	25.4 (1.7)	26.4 (0.7)	22.4 (2.0)	21.8 (0.9)
Family history of myocardial infarction, %	31.6 (1.6)	34.4 (1.7)	24.5 (1.6)	31.7 (2.1)	34.7 (1.6)	31.8 (0.8)	24.1 (1.8)	27.6 (0.9)
Family history of diabetes, %	36.7 (1.7)	53.7 (2.1)	29.1 (1.7)	45.2 (2.2)	40.9 (1.7)	45.8 (1.0)	32.2 (2.3)	38.9 (1.0)
Moderate physical activity<150 min/w, %	37.7 (1.5)	45.3 (2.1)	23.6 (1.6)	35.5 (2.2)	40.1 (1.7)	41.4 (0.9)	18.4 (2.1)	27.2 (0.8)
Healthy Eating Index-2010, points	60.0 (0.5)	57.4 (0.6)	55.5 (0.5)	54.2 (0.6)	58.7 (0.5)	59.0 (0.3)	54.9 (0.7)	56.0 (0.3)
Prevalent diabetes, %	10.7 (0.9)	26.3 (1.5)	11.6 (1.2)	32.9 (1.9)	8.9 (1.0)	20.4 (0.6)	9.6 (1.8)	19.1 (0.7)
Prevalent hypertension, %	17.7 (1.1)	39.3 (1.4)	18.7 (1.2)	45.8 (1.8)	24.0 (1.4)	28.9 (0.6)	19.7 (2.3)	30.8 (0.8)
Prevalent dyslipidemia, %	29.8 (1.3)	43.1 (1.8)	36.8 (1.7)	68.5 (2.2)	28.2 (1.6)	42.5 (0.9)	37.4 (2.1)	59.4 (1.0)
Prevalent cardiovascular disease, %	4.5 (0.7)	9.7 (1.2)	8.3 (1.0)	15.3 (1.9)	5.1 (0.8)	7.6 (0.5)	8.6 (1.2)	9.9 (0.7)
Prevalent cancer, %	4.3 (0.7)	5.2 (0.8)	1.8 (0.5)	3.5 (1.1)	4.4 (0.7)	4.7 (0.4)	0.7 (0.3)	2.8 (0.4)
Prevalent chronic obstructive pulmonary disease, %	6.9 (1.0)	13.3 (1.7)	6.3 (1.0)	7.9 (1.5)	8.5 (1.6)	7.8 (0.5)	4.3 (0.8)	4.5 (0.4)

[a] The analysis considered complex and multistage probability sampling (i.e., sampling weight, stratification, and clustering), and all estimates were standardized to the United States 2010 Decennial Census age distribution. Data are shown as mean (standard error) for continuous variables and percentage (standard error) for categorical variables.

[b] Cigarette pack years were calculated as the average daily number of used cigarettes multiply smoking years divided by 20 (number of cigarettes per pack).

© 2023 Zhang Y al. *JAMA Network Open.*

eTable 6. Distribution of Risk Factors by Body Mass Index and Waist-To-Hip Ratio[a]

	Waist-to-hip ratio (men/women) <0.90/0.85		Waist-to-hip ratio (men/women) ≥0.90/0.85	
Risk factors	BMI <35.0 kg/m^2	BMI ≥35.0 kg/m^2	BMI <35.0 kg/m^2	BMI ≥35.0 kg/m^2
Waist-to-hip ratio	0.83 (0.00)	0.82 (0.00)	0.94 (0.00)	0.97 (0.00)
Men	0.87 (0.00)	0.87 (0.01)	0.97 (0.00)	1.02 (0.00)
Women	0.81 (0.00)	0.81 (0.00)	0.92 (0.00)	0.94 (0.00)
BMI, kg/m^2	25.9 (0.1)	39.9 (0.4)	28.5 (0.1)	40.0 (0.1)
Men	25.0 (0.1)	37.3 (0.4)	28.7 (0.1)	39.4 (0.2)
Women	26.3 (0.1)	40.2 (0.5)	28.3 (0.1)	40.3 (0.2)
Waist circumference, cm	85.2 (0.2)	107.2 (1.0)	97.6 (0.1)	120.4 (0.4)
Men	86.3 (0.3)	113.1 (2.5)	99.2 (0.2)	123.4 (0.6)
Women	84.1 (0.3)	106.1 (1.0)	96.1 (0.2)	118.6 (0.4)
Hip circumference, cm	102.6 (0.3)	131.5 (1.5)	103.5 (0.1)	124.3 (0.4)
Men	99.7 (0.3)	131.9 (4.8)	102.7 (0.2)	121.7 (0.6)
Women	104.4 (0.3)	131.0 (1.4)	104.2 (0.2)	125.9 (0.4)
Age, years	33.5 (0.3)	43.4 (1.9)	43.9 (0.3)	41.2 (0.5)
Men, %	43.7 (1.3)	11.1 (2.5)	49.6 (0.8)	38.6 (1.6)
Single, %	34.9 (1.1)	33.0 (3.1)	29..8 (0.8)	32.7 (1.3)
Yearly household income <$30,000, %	58.0 (1.5)	74.0 (3.6)	61.9 (1.0)	63.7 (1.6)
<High school/GED, %	27.7 (1.3)	31.2 (4.5)	34.9 (0.9)	34.8 (1.4)
Unemployed, %	39.4 (1.3)	49.5 (4.4)	38.2 (0.8)	45.0 (1.4)
No current health insurance, %	47.1 (1.6)	46.7 (4.8)	49.5 (1.0)	42.8 (1.5)
Reside in US <10 years, %	32.4 (1.7)	23.5 (3.4)	27.1 (1.0)	17.0 (1.3)
Preferred to speak in Spanish, %	78.5 (1.1)	64.8 (5.9)	78.9 (0.9)	66.6 (1.7)
Cigarette pack years, pack-years[b]	4.8 (0.5)	2.9 (0.6)	5.8 (0.2)	5.9 (0.5)
Former or heavy alcohol drinking, %	31.7 (1.4)	44.0 (4.3)	36.4 (0.8)	40.0 (1.5)
Sleep of <6 or >9 h/d, %	23.3 (1.1)	30.6 (4.2)	24.1 (0.7)	25.1 (1.3)
Family history of myocardial infarction, %	30.4 (1.2)	37.2 (3.6)	29.1 (0.7)	33.2 (1.3)
Family history of diabetes, %	36.1 (1.5)	47.5 (4.1)	40.6 (0.8)	50.9 (1.6)
Moderate physical activity<150 min/w, %	29.6 (1.3)	50.2 (4.8)	33.4 (0.7)	40.6 (1.5)
Healthy Eating Index-2010, points	57.2 (0.4)	58.0 (1.2)	58.0 (0.3)	55.9 (0.4)
Fasting blood glucose, mg/dL	95.6 (0.5)	98.8 (1.3)	103.7 (0.5)	111.2 (1.4)
2-hour blood glucose after OGTT, mg/dL	112.8 (1.3)	135.4 (3.2)	122.8 (0.7)	136.0 (1.5)
Glycosylated hemoglobin, %	5.5 (0.0)	5.8 (0.1)	5.8 (0.0)	6.2 (0.0)
Total cholesterol, mg/dL	194.3 (1.1)	196.5 (3.1)	199.1 (0.6)	192.4 (1.0)
Triglyceride, mg/dL	105.3 (1.7)	115.7 (3.2)	144.7 (1.8)	148.1 (2.5)
High-density lipoprotein cholesterol, mg/dL	53.7 (0.3)	49.4 (1.1)	47.7 (0.2)	44.5 (0.3)
Low-density lipoprotein cholesterol, mg/dL	119.6 (1.0)	123.9 (2.8)	123.4 (0.6)	118.8 (0.8)

© 2023 Zhang Y al. *JAMA Network Open.*

Systolic blood pressure, mm Hg	119.6 (0.4)	122.8 (1.5)	121.3 (0.2)	123.3 (0.6)
Diastolic blood pressure, mm Hg	70.3 (0.3)	77.1 (0.9)	72.4 (0.2)	77.3 (0.4)
Pulse rate, per min	63.3 (0.2)	68.4 (0.9)	65.2 (0.2)	69.1 (0.4)
Prevalent diabetes, %	8.8 (1.0)	13.4 (2.5)	17.6 (0.5)	30.8 (1.2)
Prevalent hypertension, %	20.4 (1.0)	38.6 (3.0)	27.3 (0.5)	41.9 (1.2)
Prevalent dyslipidemia, %	33.7 (1.3)	36.0 (4.9)	49.7 (0.8)	54.7 (1.5)
Prevalent cardiovascular disease, %	6.2 (0.8)	4.9 (1.5)	7.8 (0.4)	12.4 (1.1)
Prevalent cancer, %	3.0 (0.6)	4.6 (1.8)	3.7 (0.3)	4.7 (0.6)
Prevalent chronic obstructive pulmonary disease, %	5.8 (0.7)	14.3 (3.7)	5.3 (0.3)	10.9 (1.0)

Abbreviation: BMI, body mass index.

[a] The analysis considered complex and multistage probability sampling (i.e., sampling weight, stratification, and clustering), and all estimates were standardized to the United States 2010 Decennial Census age distribution. Data are shown as mean (standard error) for continuous variables and percentage (standard error) for categorical variables.

[b] Cigarette pack years were calculated as the average daily number of used cigarettes multiply smoking years divided by 20 (number of cigarettes per pack).

© 2023 Zhang Y al. *JAMA Network Open.*

eTable 7. Prevalence of Groups with Different Body Mass Index and Waist-to-Hip Ratio

Body mass index	Waist-to-hip ratio (men/women)	
	<0.90/0.85	≥0.90/0.85
18.5-24.9 kg/m^2	11.3%	10.4%[a]
25.0-29.9 kg/m^2	8.9%[b]	29.1%
30.0-34.5 kg/m^2	3.0%[b]	22.2%
≥35.0 kg/m^2	1.9%[b]	13.2%

[a] The group would be misclassified as normal weight based on body mass index alone.
[b] The groups would be misclassified as overweight or obesity based on body mass index alone.

© 2023 Zhang Y al. *JAMA Network Open.*
