# Supplementary material for: General or Central Obesity and Mortality Among US Hispanic and Latino Adults
Source: JAMA Netw Open. 2024 Jan 16;7(1):e2351070. doi: 10.1001/jamanetworkopen.2023.51070 (PMC10792478; doi:10.1001/jamanetworkopen.2023.51070)
Supplement: Supplement 2. — Data Sharing Statement [file jamanetwopen-e2351070-s002.pdf]

Data Sharing Statement

Zhang. General or Central Obesity and Mortality Among US Hispanic and Latino Adults. *JAMA Netw Open*. Published January 16, 2024. doi:10.1001/jamanetworkopen.2023.51070

Data
Data available: Yes
Data types: Deidentified participant data
How to access data: The datasets generated during and/or analyzed in the current study are available from the corresponding author upon reasonable request.
When available: With publication

Supporting Documents
Document types: Statistical/analytic code
How to access documents: The datasets generated during and/or analyzed in the current study are available from the corresponding author upon reasonable request.
When available: With publication

Additional Information
Who can access the data: Researchers whose proposed use of the data has been approved.
Types of analyses: Projects approved by the Hispanic Community Health Study / Study of Latinos Committee.
Mechanisms of data availability: With investigator support and after approval of a proposal.
